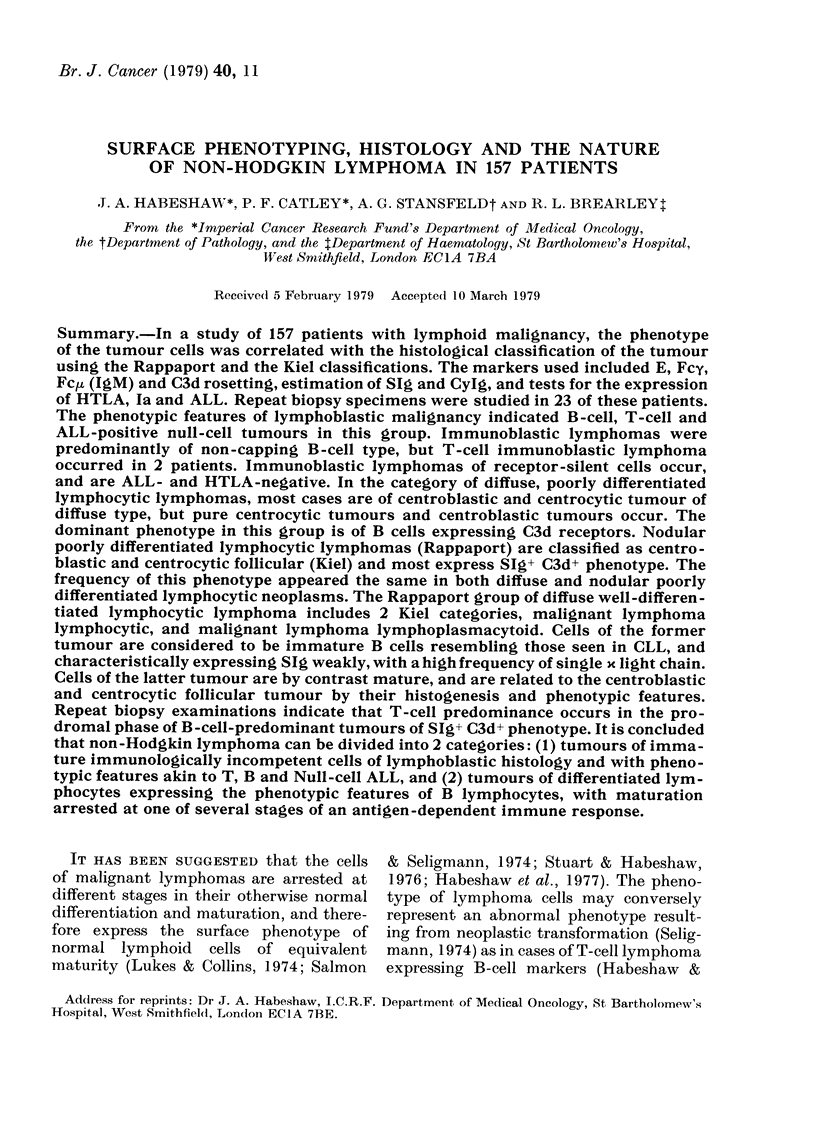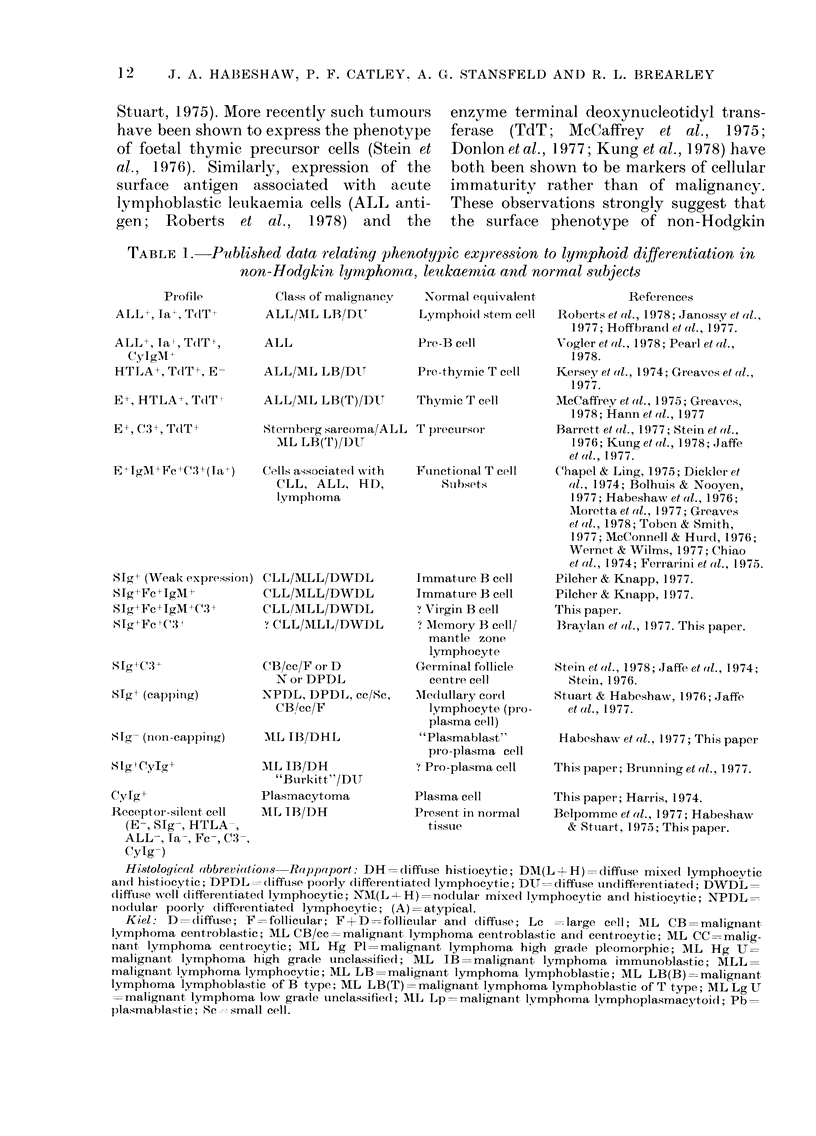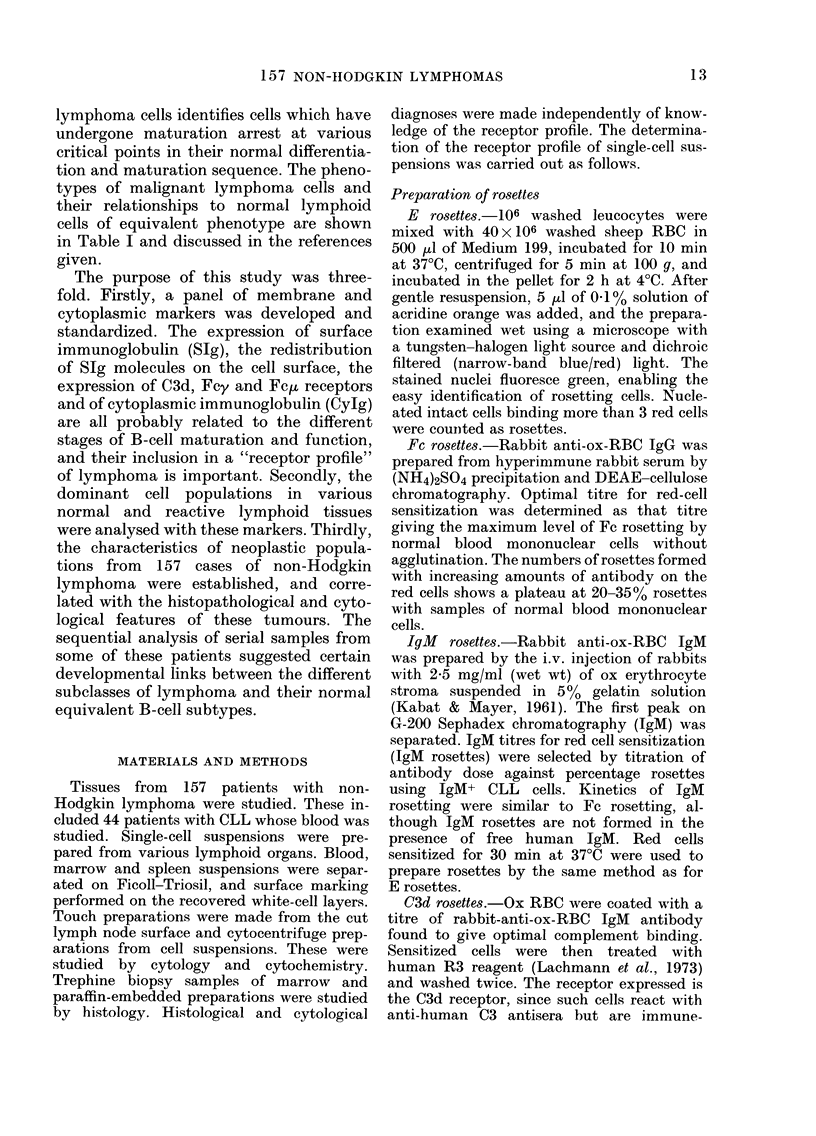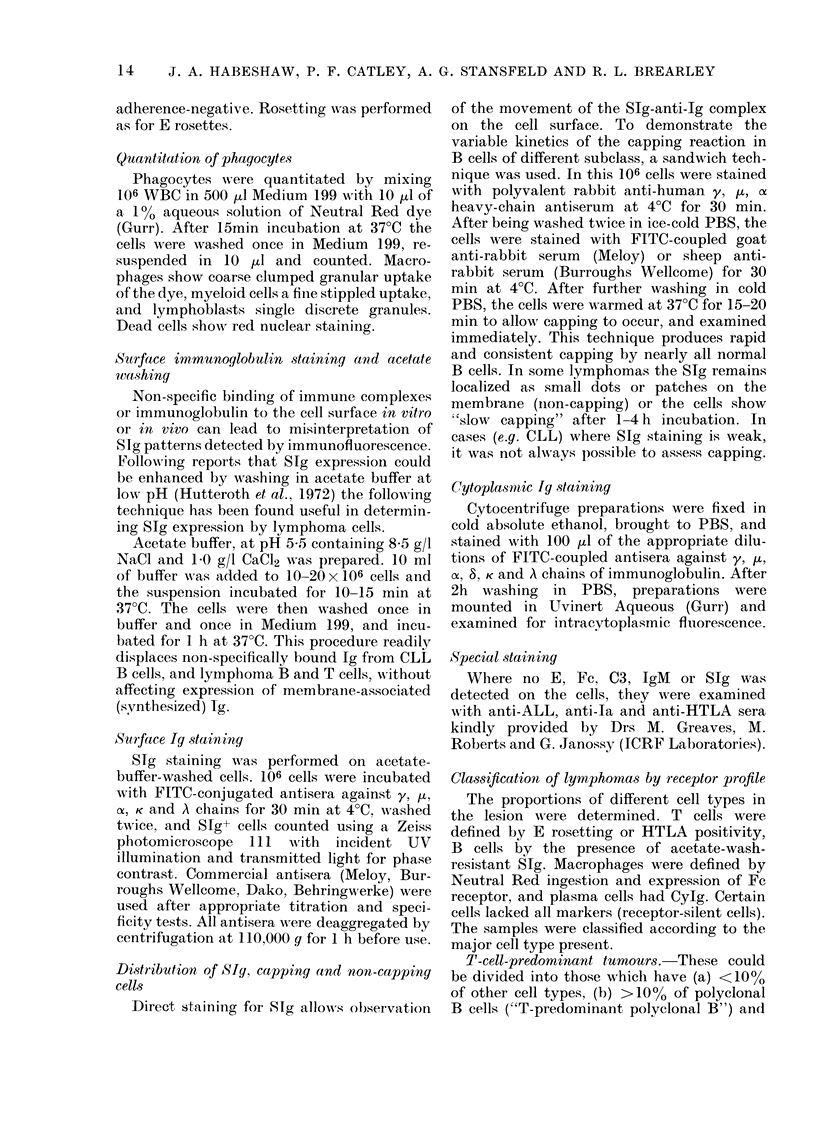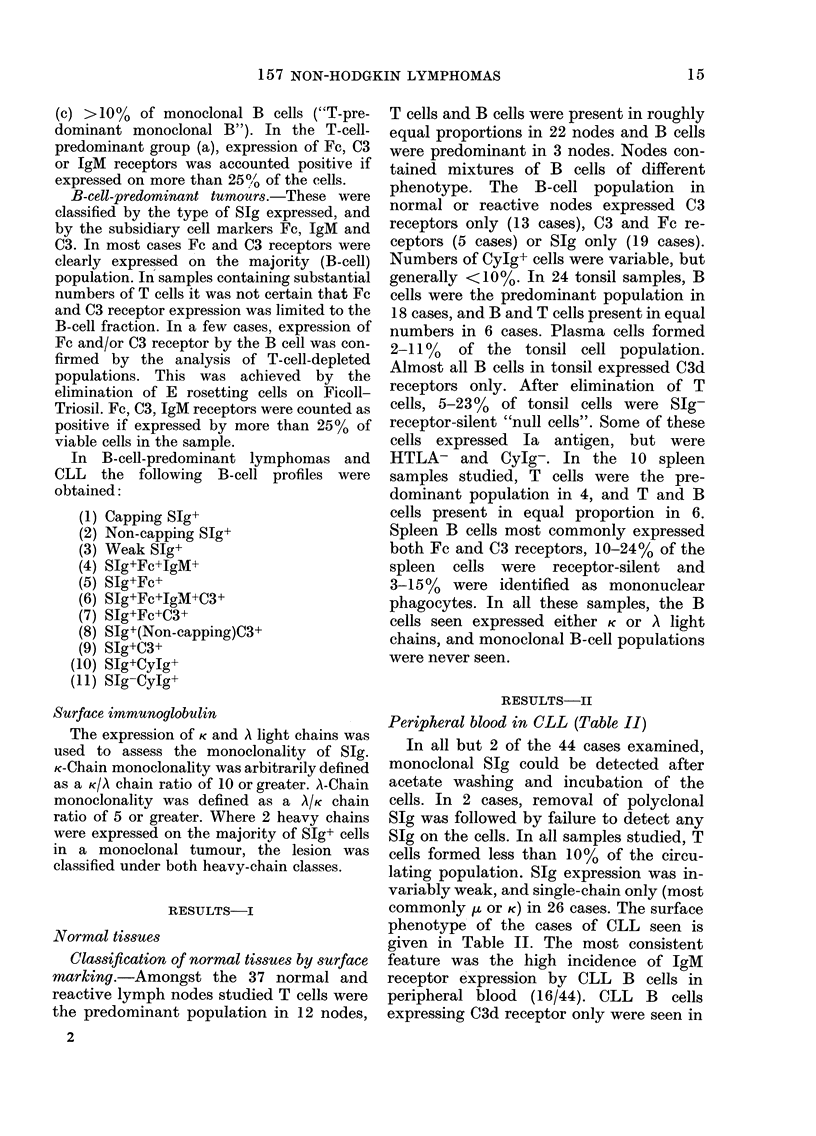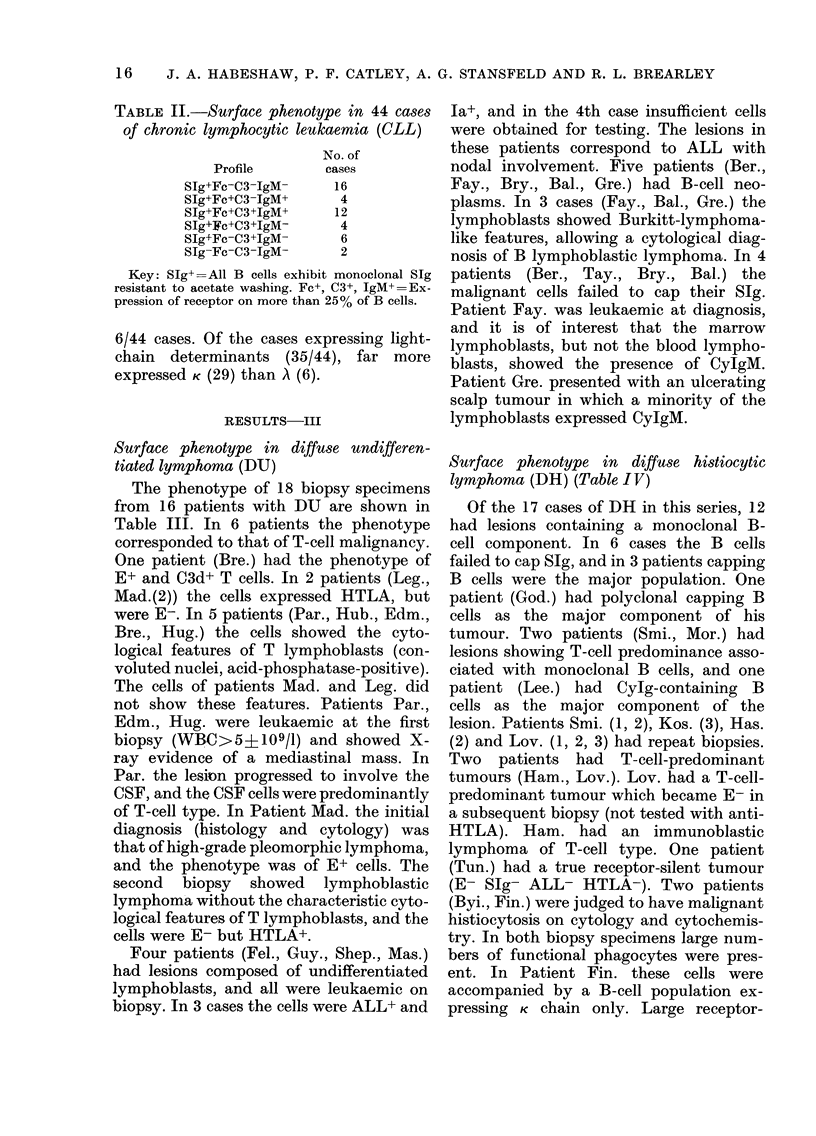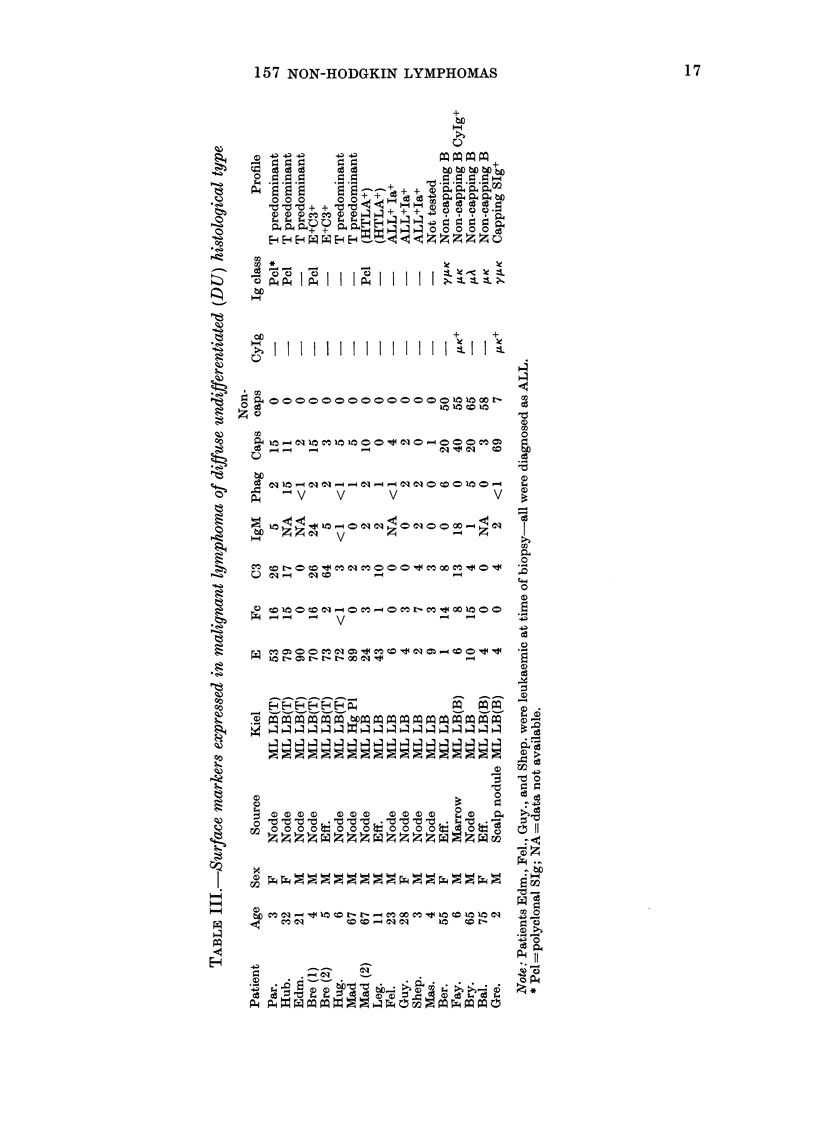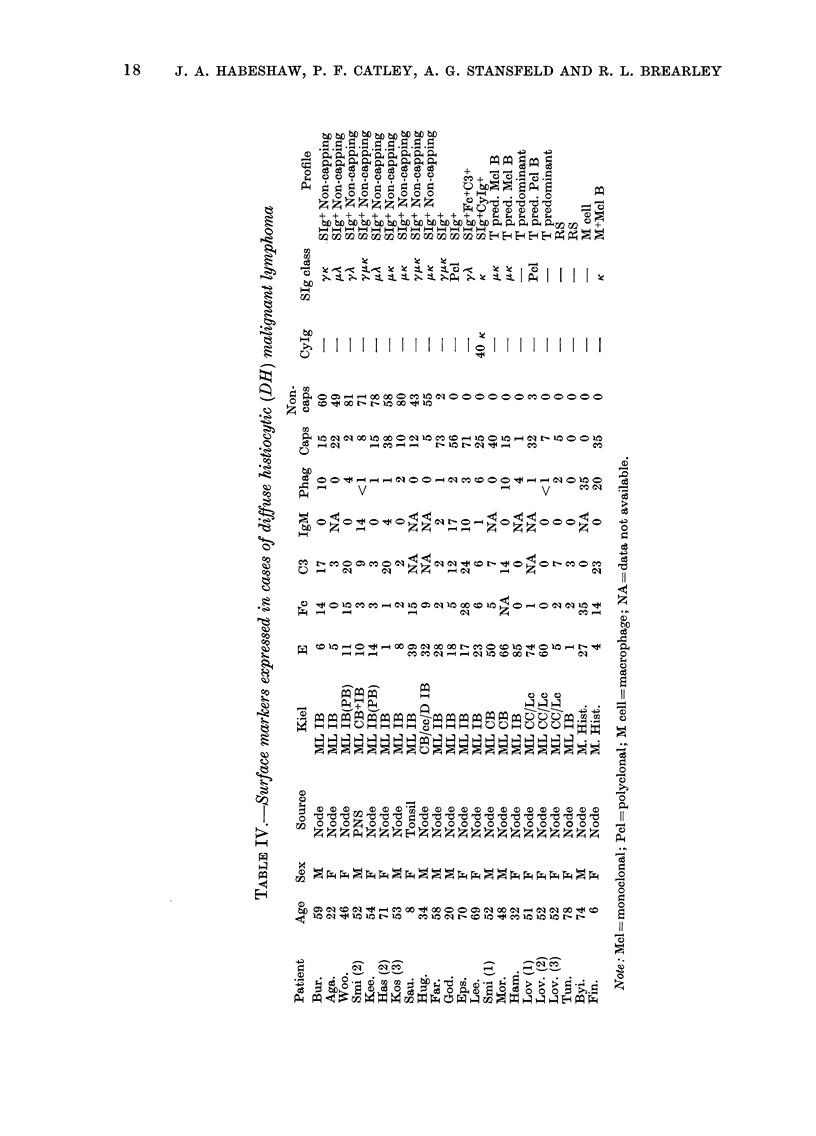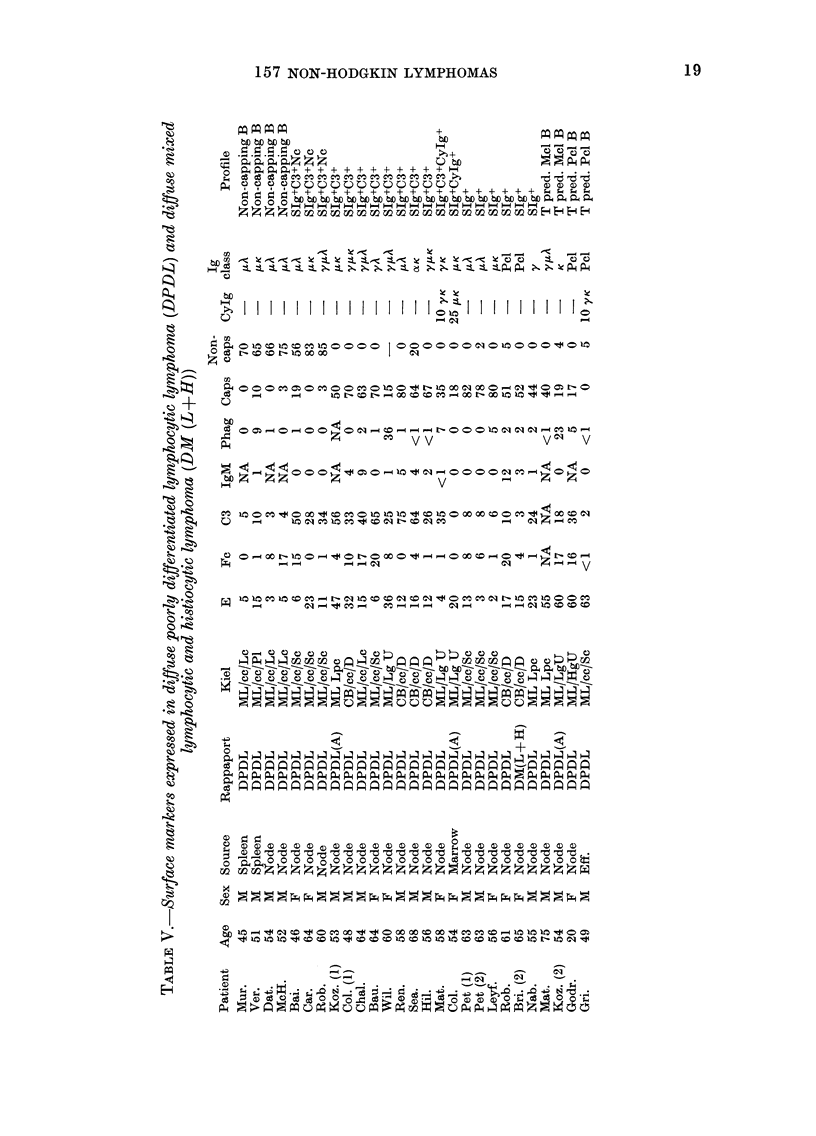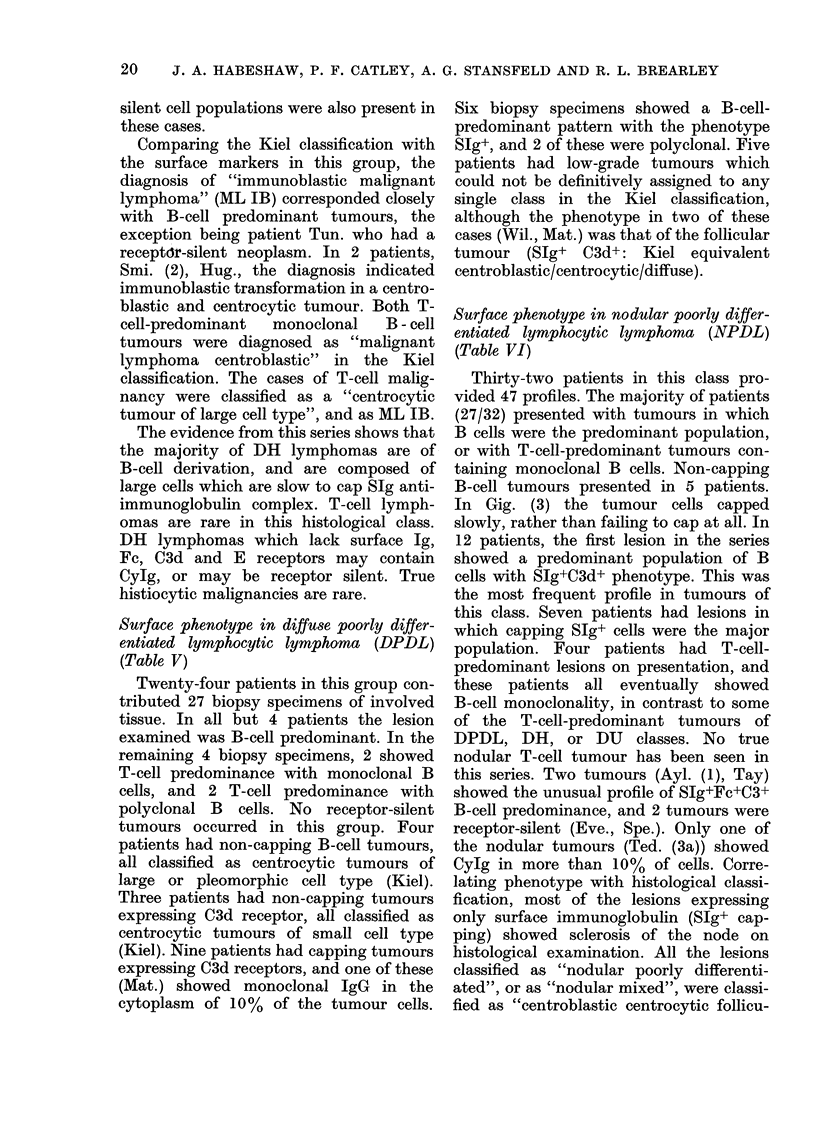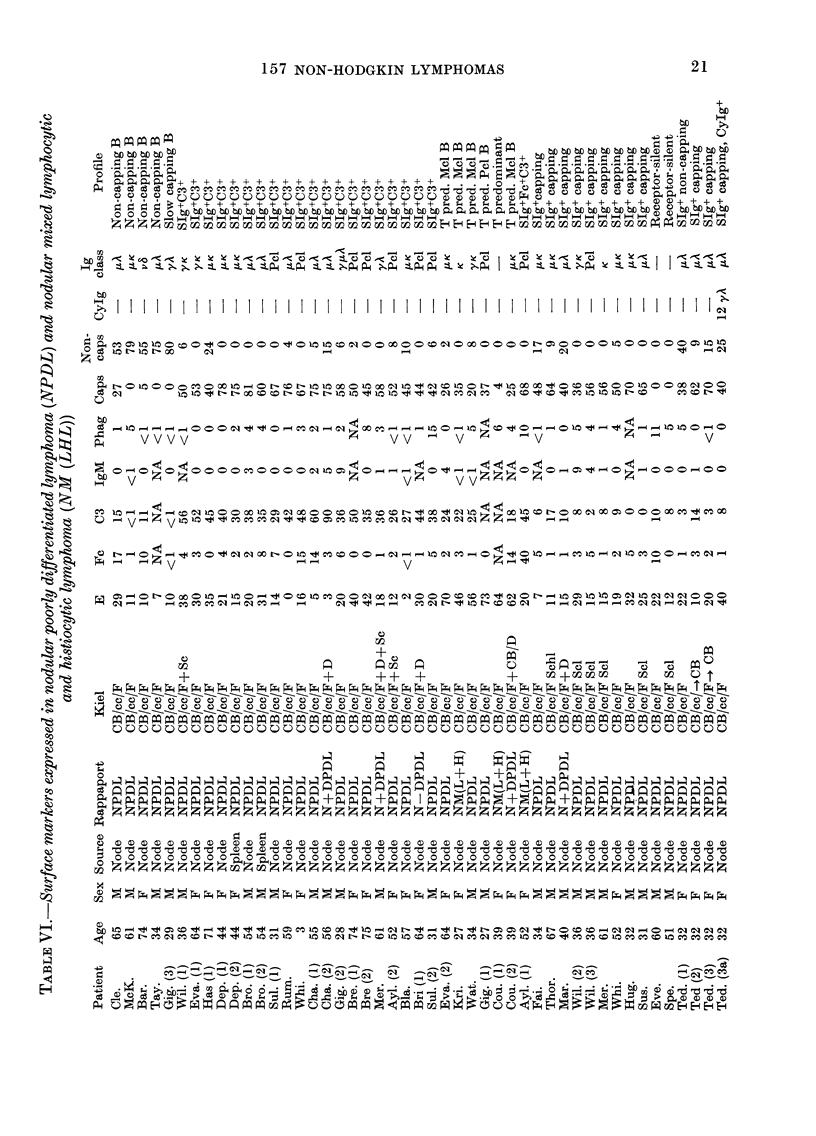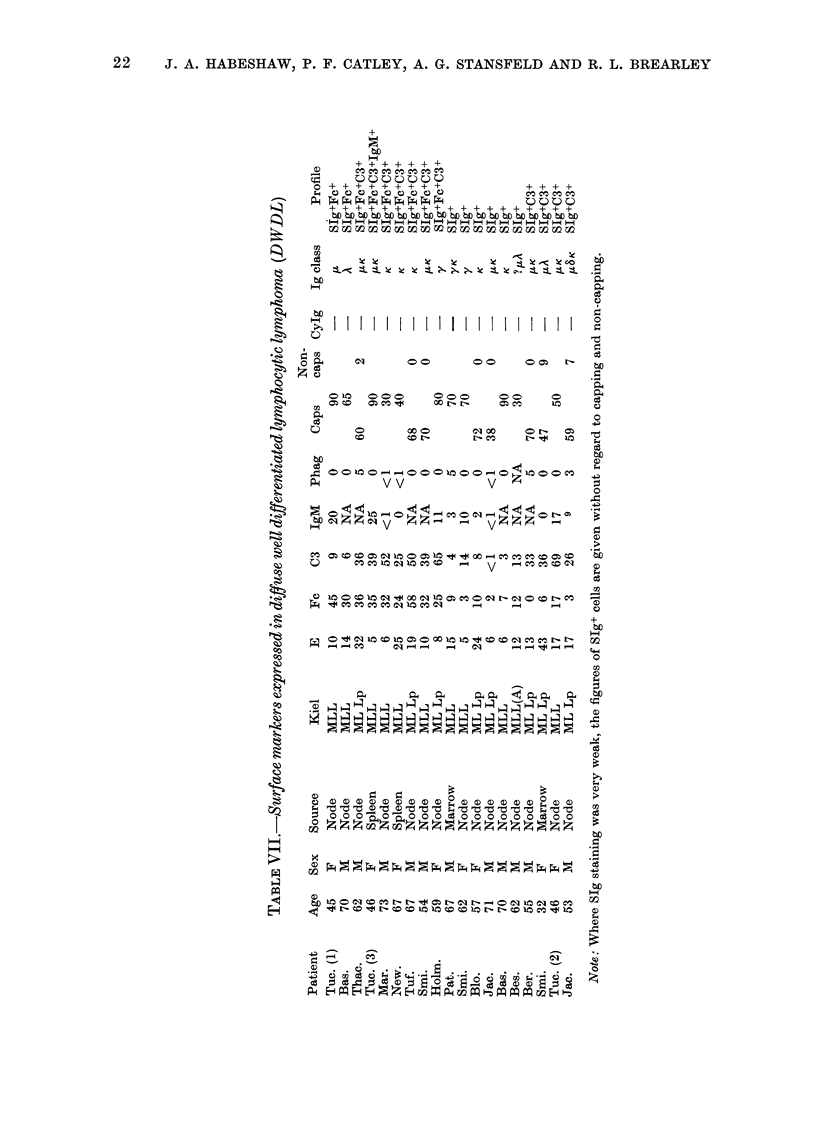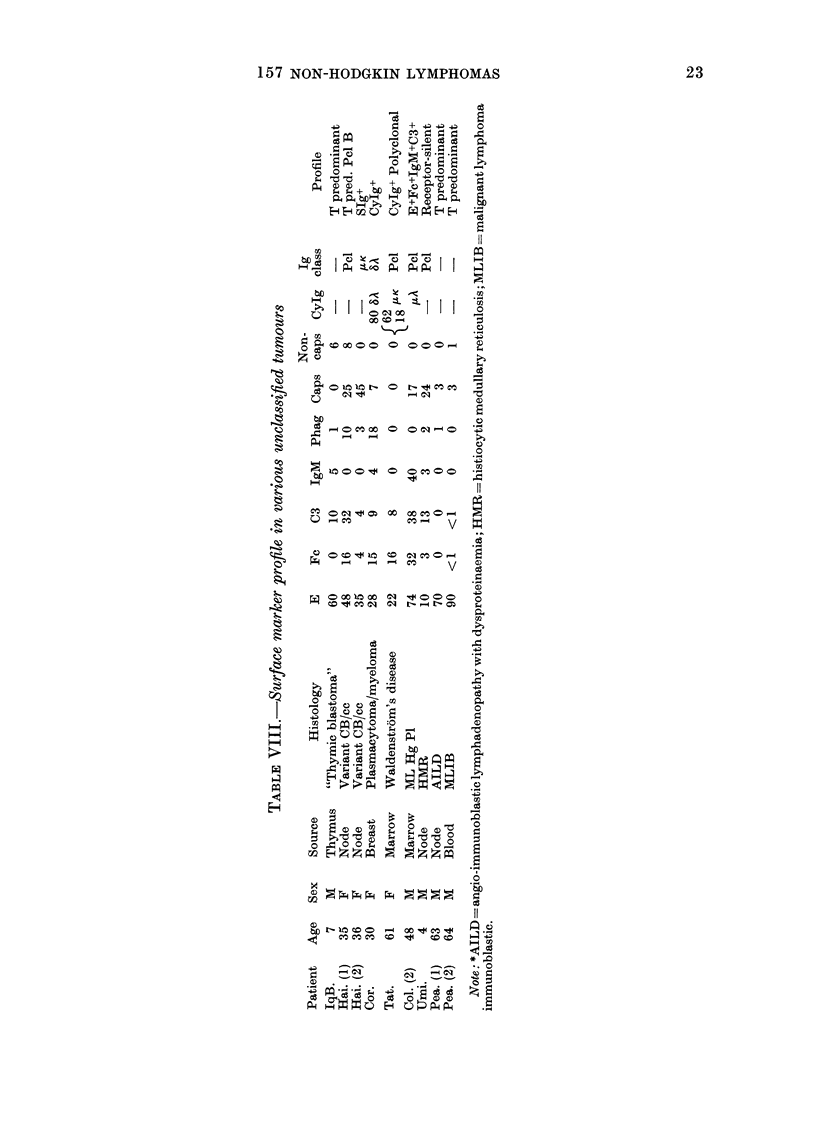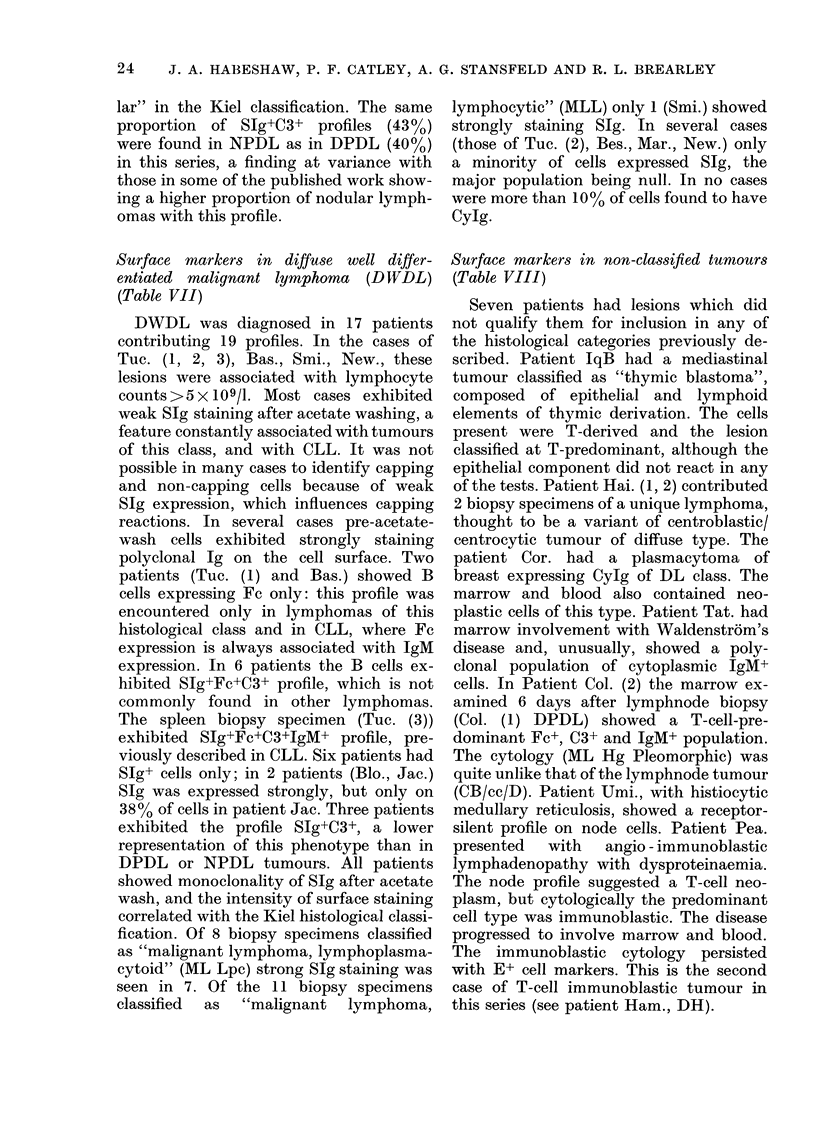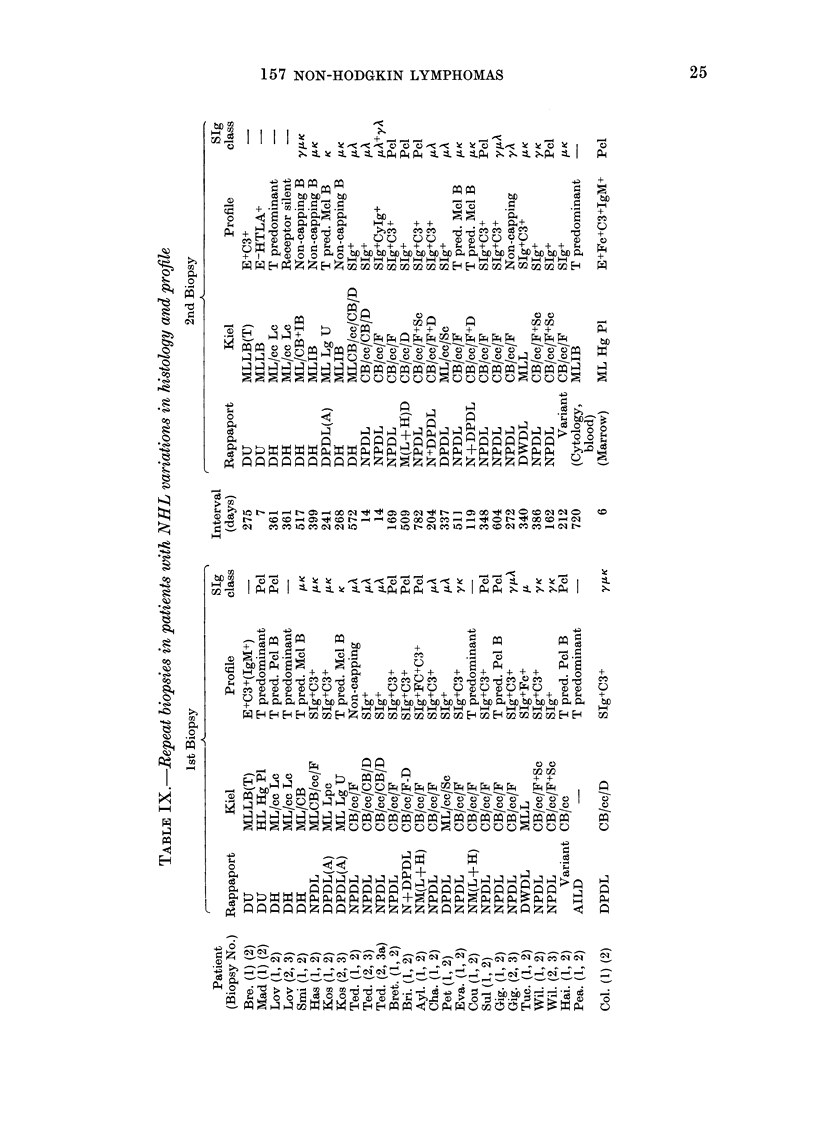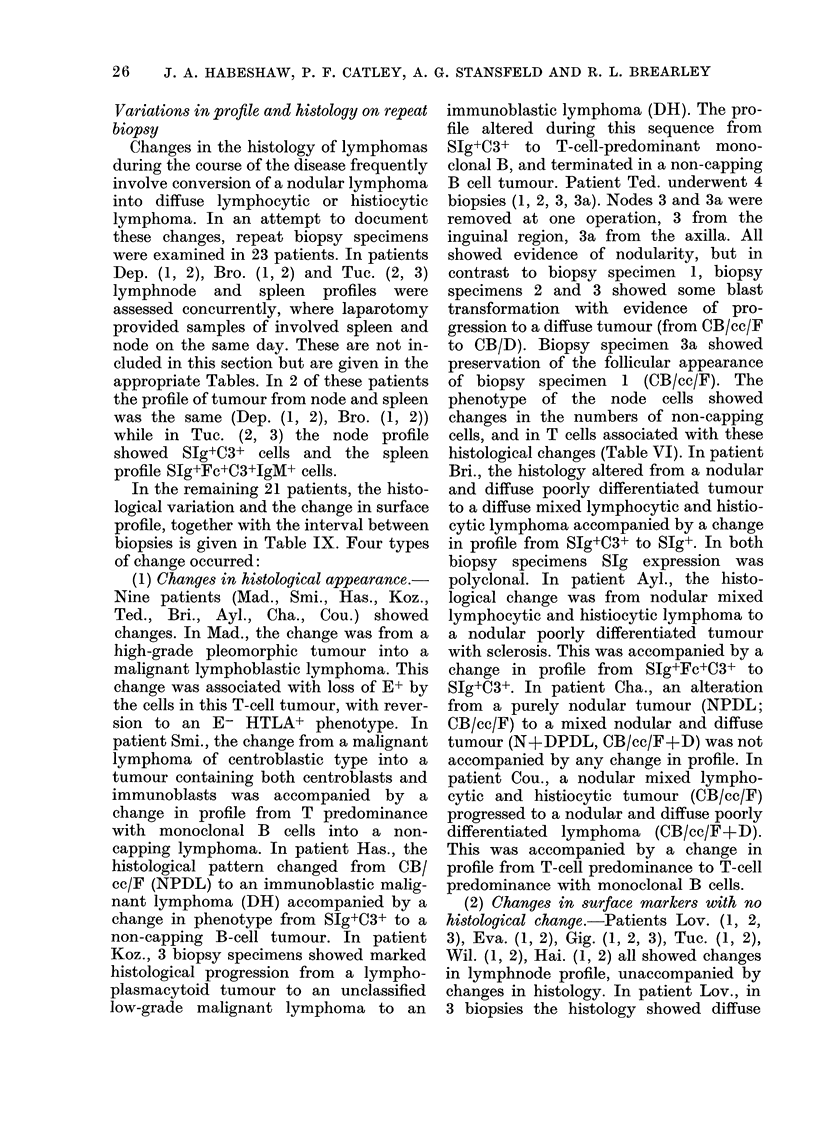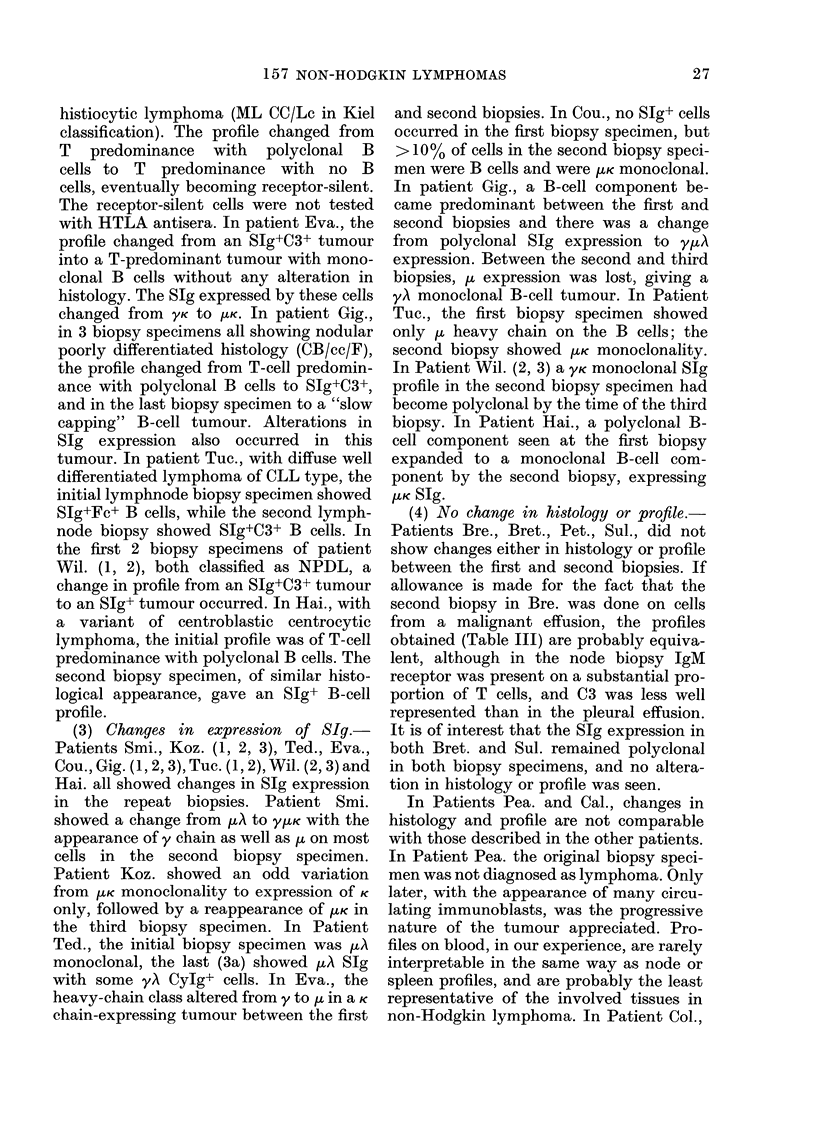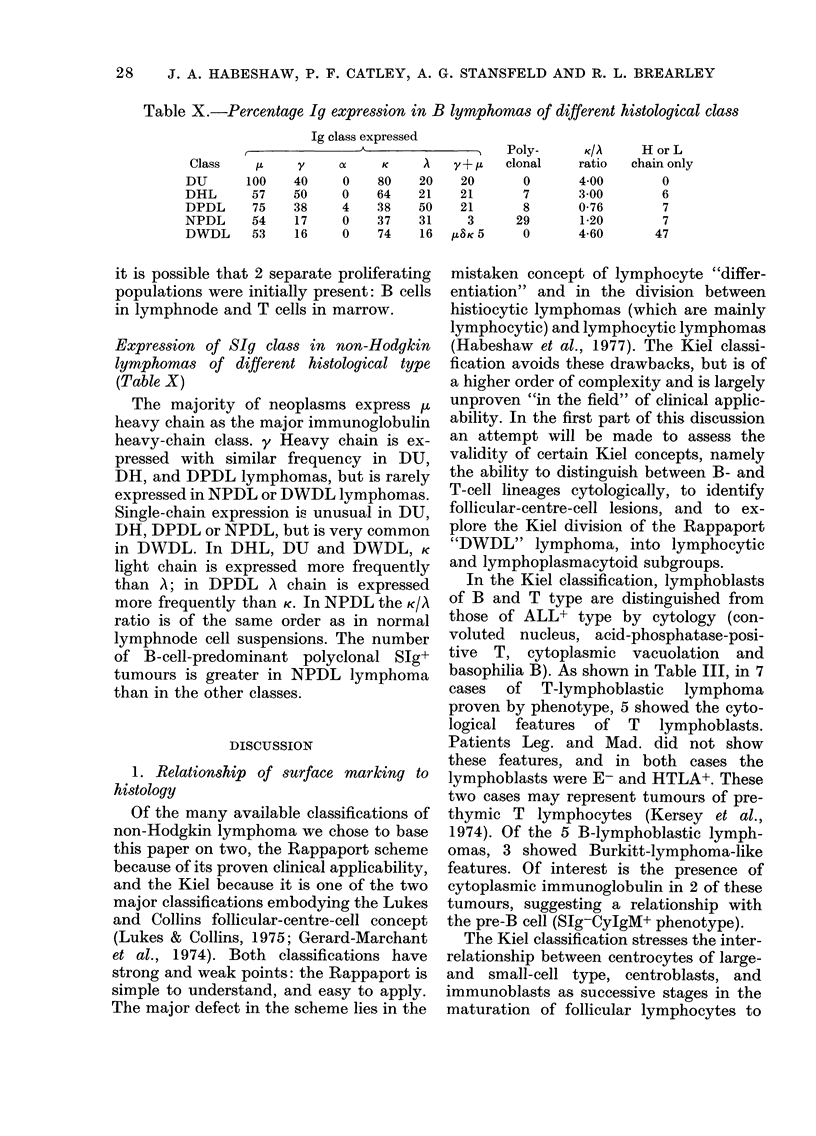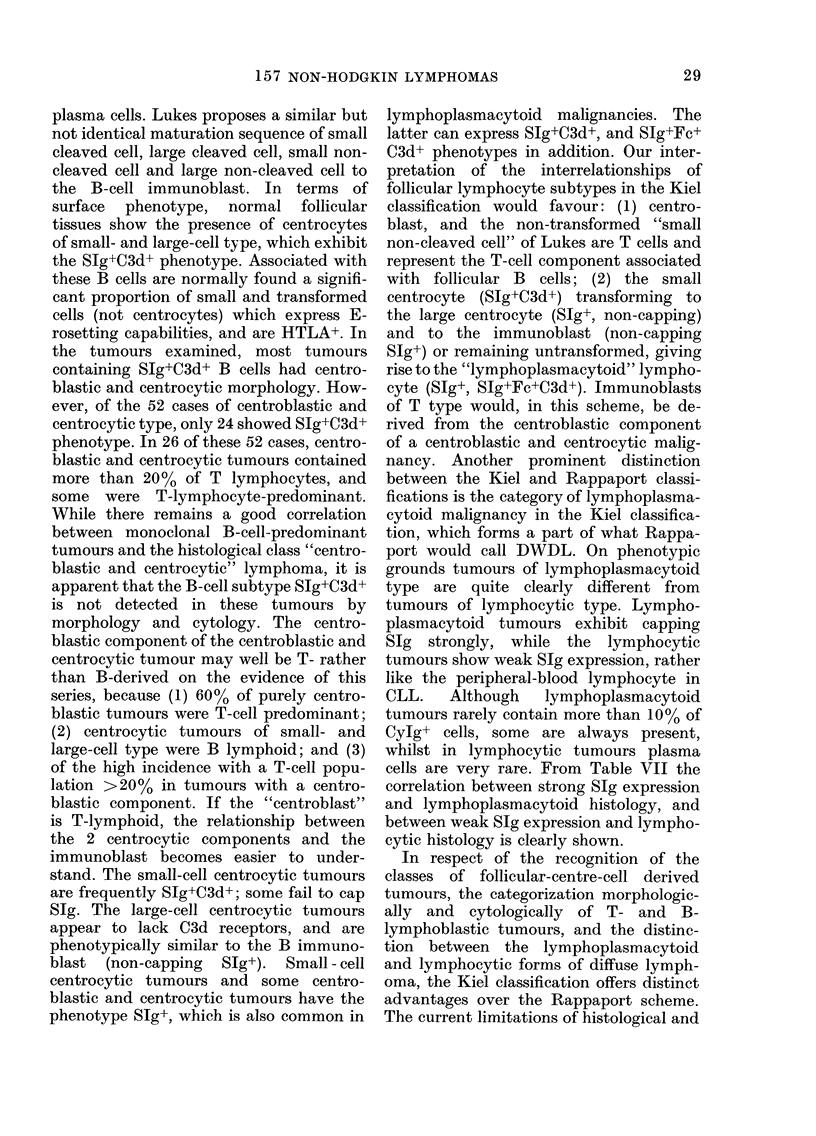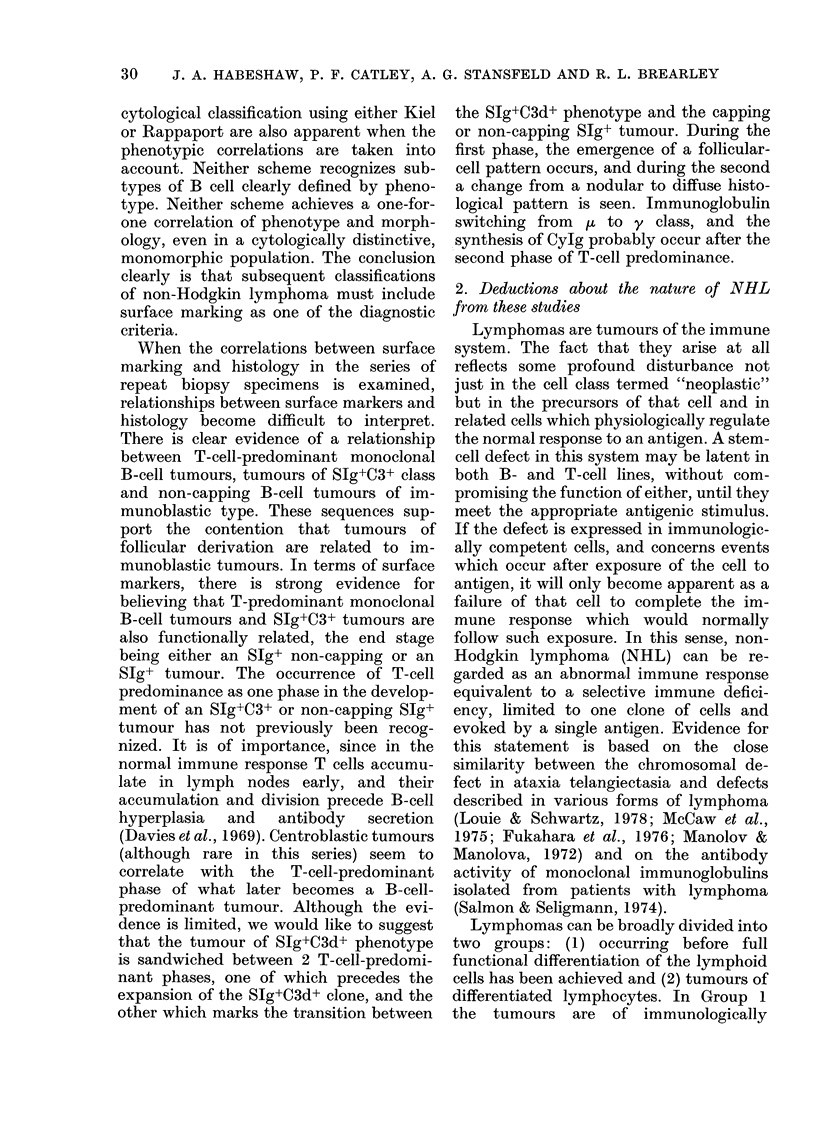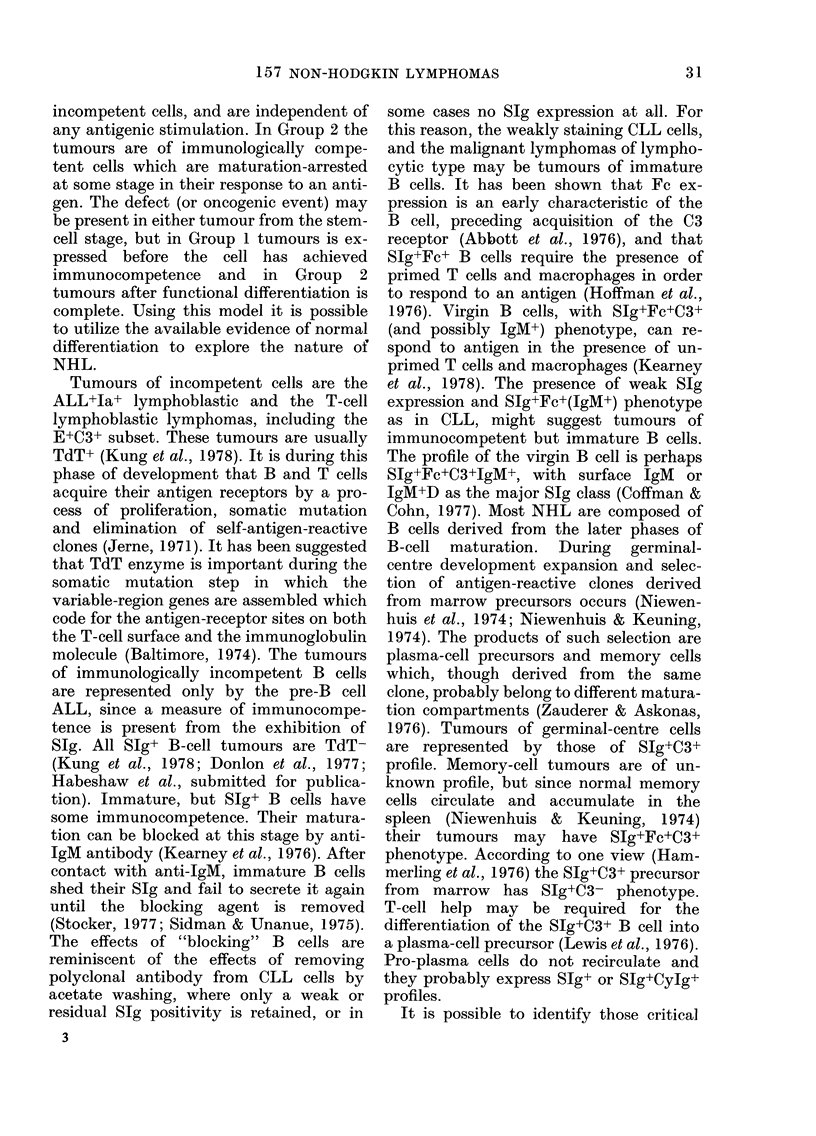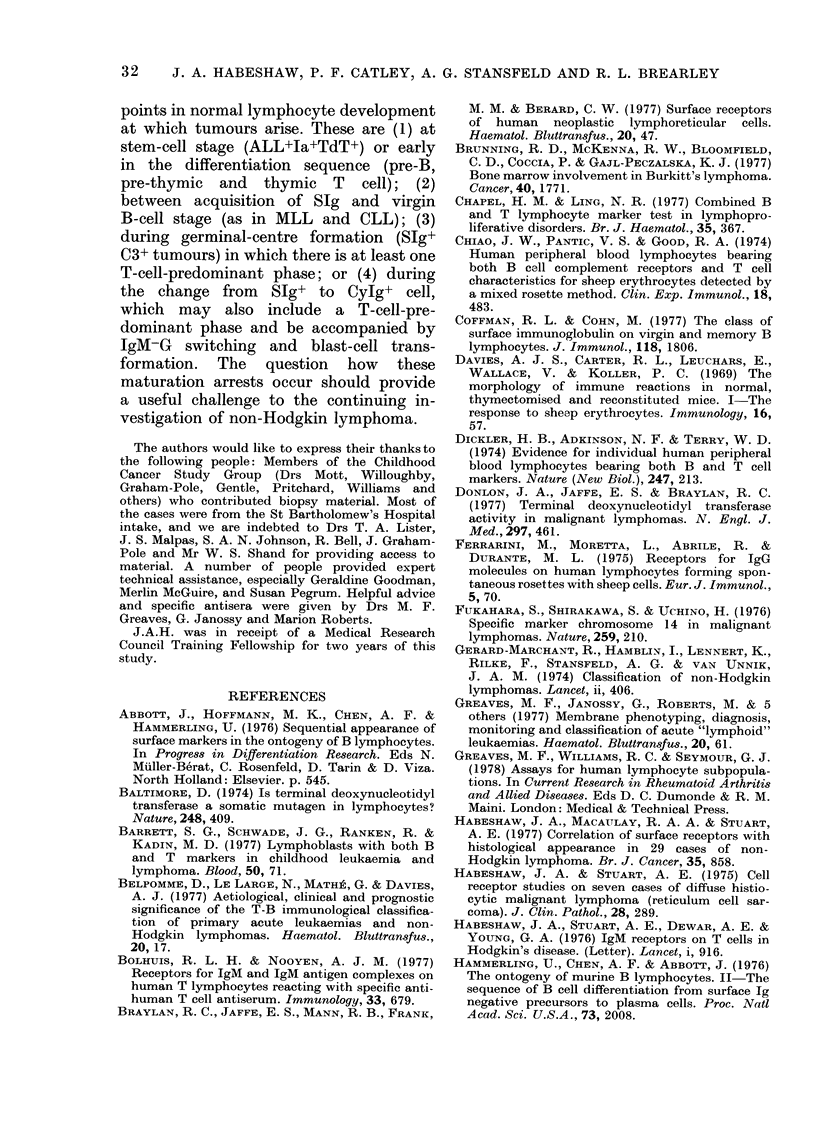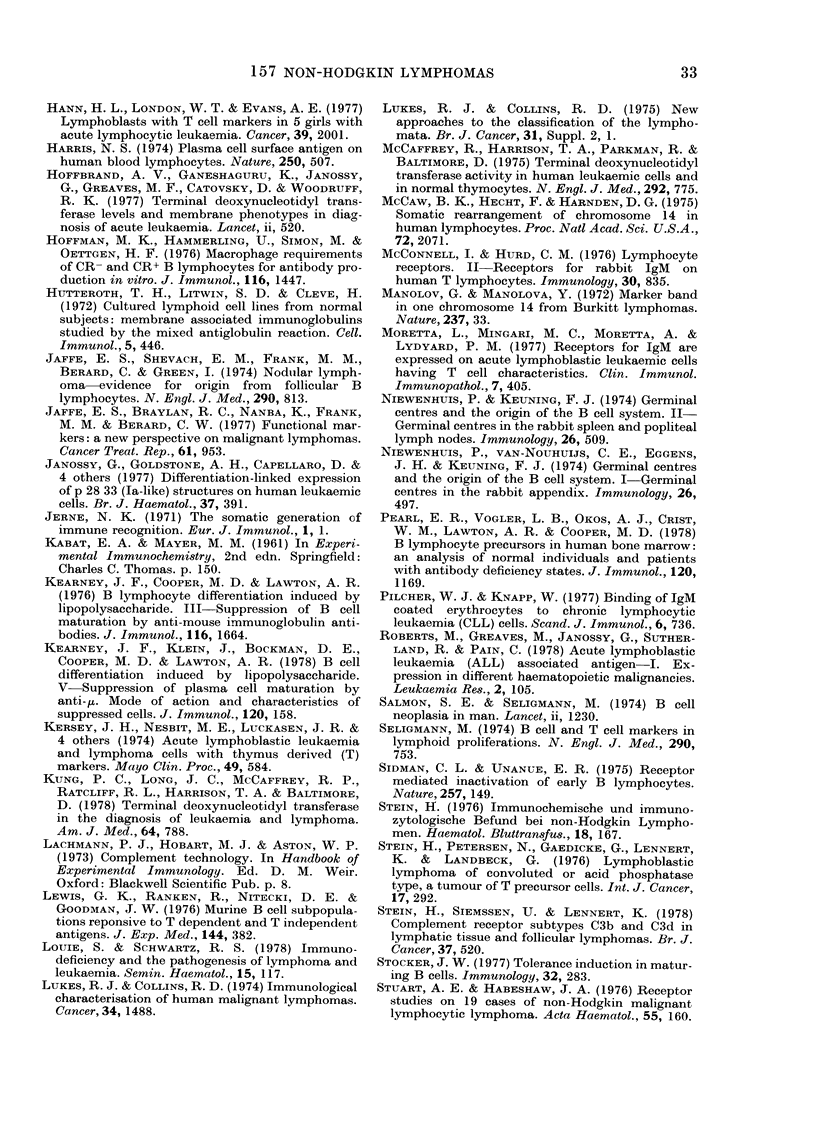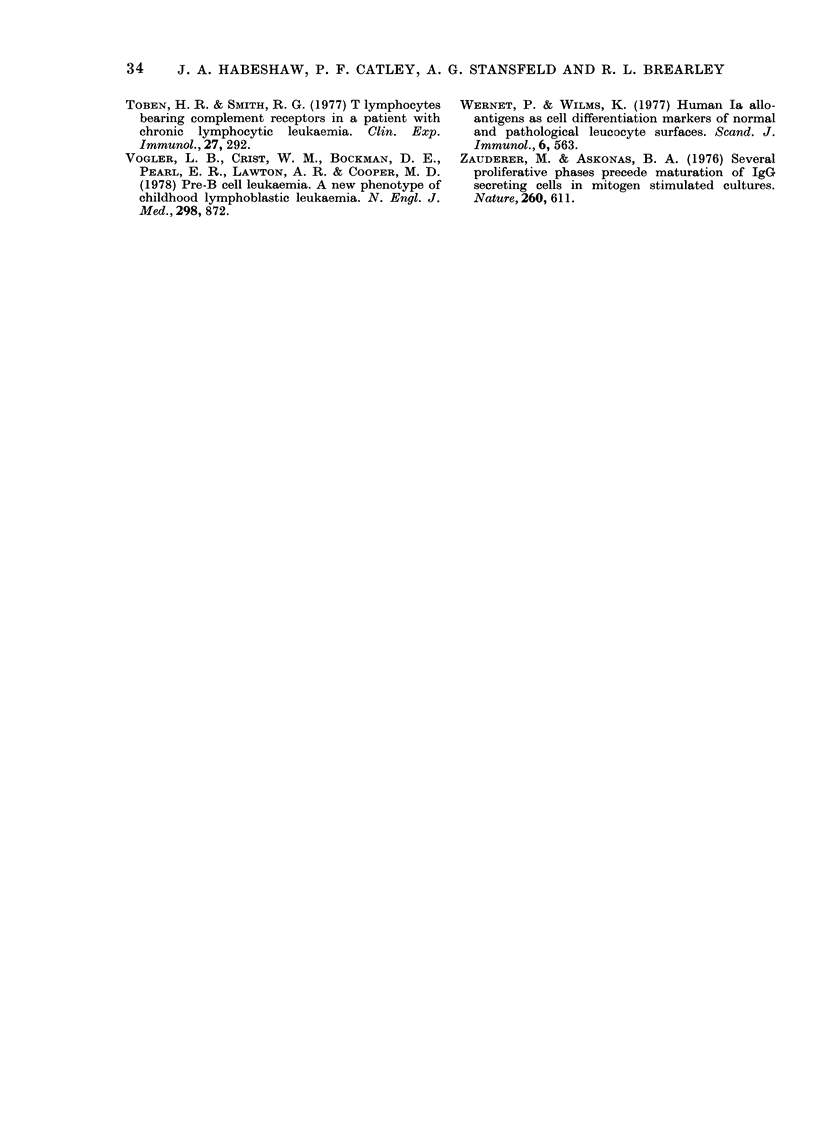# Surface phenotyping, histology and the nature of non-Hodgkin lymphoma in 157 patients.

**DOI:** 10.1038/bjc.1979.137

**Published:** 1979-07

**Authors:** J. A. Habeshaw, P. F. Catley, A. G. Stansfeld, R. L. Brearley

## Abstract

In a study of 157 patients with lymphoid malignancy, the phenotype of the tumour cells was correlated with the histological classification of the tumour using the Rappaport and the Kiel classifications. The markers used included E, Fc gamma, Fc micron (IgM) and C3d rosetting, estimation of SIg and CyIg, and tests for the expression of HTLA, Ia and ALL. Repeat biopsy specimens were studied in 23 of these patients. The phenotypic features of lymphoblastic malignancy indicated B-cell, T-cell and ALL-positive null-cell tumours in this group. Immunoblastic lymphomas were predominantly of non-capping B-cell type, but T-cell immunoblastic lymphoma occurred in 2 patients. Immunoblastic lymphomas of receptor-silent cells occur, and are ALL- and HTLA-negative. In the category of diffuse, poorly differentiated lymphocytic lymphomas, most cases are of centroblastic and centrocytic tumour of diffuse type, but pure centrocytic tumours and centroblastic tumours occur. The dominant phenotype in this group is of B cells expressing C3d receptors. Nodular poorly differentiated lymphocytic lymphomas (Rappaport) are classified as centroblastic and centrocytic follicular (Kiel) and most express SIg+ C3d+ phenotype. The frequency of this phenotype appeared the same in both diffuse and nodular poorly differentiated lymphocytic neoplasms. The Rappaport group of diffuse well-differentiated lymphocytic lymphoma includes 2 Kiel categories, malignant lymphoma lymphocytic, and malignant lymphoma lymphoplasmacytoid. Cells of the former tumour were considered to be immature B cells resembling those seen in CLL, and characteristically expressing SIg weakly, with a high frequency of single kappa light chain. Cells of the latter tumour are by contrast mature, and are related to the centroblastic and centrocytic follicular tumour by their histogenesis and phenotypic features. Repeat biopsy examinations indicate that T-cell predominance occurs in the prodromal phase of B-cell-predominant tumours of SIg+ C3d+ phenotype. It is concluded that non-Hodgkin lymphoma can be divided into 2 categories: (1) tumours of immature immunologically incompetent cells of lymphoblastic histology and with phenotypic features akin to T, B and Null-cell ALL, and (2) tumours of differentiated lymphocytes expressing the phenotypic features of B lymphocytes, with maturation arrested at one of several stages of an antigen-dependent immune response.


*Br. J. Cancer* (1979) **40**, 11

### SURFACE PHENOTYPING, HISTOLOGY AND THE NATURE OF NON-HODGKIN LYMPHOMA IN 157 PATIENTS

J. A. HABESHAW*, P. F. CATLEY*, A. G. STANSFELD† AND R. L. BREARLEY‡

*From the *Imperial Cancer Research Fund's Department of Medical Oncology, the †Department of Pathology, and the ‡Department of Haematology, St Bartholomew's Hospital, West Smithfield, London EC1A 7BA*

Received 5 February 1979 Accepted 10 March 1979

**Summary.—In a study of 157 patients with lymphoid malignancy, the phenotype of the tumour cells was correlated with the histological classification of the tumour using the Rappaport and the Kiel classifications. The markers used included E, Fcγ, Fcμ (IgM) and C3d rosetting, estimation of SIg and CyIg, and tests for the expression of HTLA, Ia and ALL. Repeat biopsy specimens were studied in 23 of these patients. The phenotypic features of lymphoblastic malignancy indicated B-cell, T-cell and ALL-positive null-cell tumours in this group. Immunoblastic lymphomas were predominantly of non-capping B-cell type, but T-cell immunoblastic lymphoma occurred in 2 patients. Immunoblastic lymphomas of receptor-silent cells occur, and are ALL- and HTLA-negative. In the category of diffuse, poorly differentiated lymphocytic lymphomas, most cases are of centroblastic and centrocytic tumour of diffuse type, but pure centrocytic tumours and centroblastic tumours occur. The dominant phenotype in this group is of B cells expressing C3d receptors. Nodular poorly differentiated lymphocytic lymphomas (Rappaport) are classified as centroblastic and centrocytic follicular (Kiel) and most express $SIg^+$ $C3d^+$ phenotype. The frequency of this phenotype appeared the same in both diffuse and nodular poorly differentiated lymphocytic neoplasms. The Rappaport group of diffuse well-differentiated lymphocytic lymphoma includes 2 Kiel categories, malignant lymphoma lymphocytic, and malignant lymphoma lymphoplasmacytoid. Cells of the former tumour are considered to be immature B cells resembling those seen in CLL, and characteristically expressing SIg weakly, with a high frequency of single κ light chain. Cells of the latter tumour are by contrast mature, and are related to the centroblastic and centrocytic follicular tumour by their histogenesis and phenotypic features. Repeat biopsy examinations indicate that T-cell predominance occurs in the prodromal phase of B-cell-predominant tumours of $SIg^+$ $C3d^+$ phenotype. It is concluded that non-Hodgkin lymphoma can be divided into 2 categories: (1) tumours of immature immunologically incompetent cells of lymphoblastic histology and with phenotypic features akin to T, B and Null-cell ALL, and (2) tumours of differentiated lymphocytes expressing the phenotypic features of B lymphocytes, with maturation arrested at one of several stages of an antigen-dependent immune response.**

IT HAS BEEN SUGGESTED that the cells of malignant lymphomas are arrested at different stages in their otherwise normal differentiation and maturation, and therefore express the surface phenotype of normal lymphoid cells of equivalent maturity (Lukes & Collins, 1974; Salmon & Seligmann, 1974; Stuart & Habeshaw, 1976; Habeshaw *et al.*, 1977). The phenotype of lymphoma cells may conversely represent an abnormal phenotype resulting from neoplastic transformation (Seligmann, 1974) as in cases of T-cell lymphoma expressing B-cell markers (Habeshaw &

Address for reprints: Dr J. A. Habeshaw, I.C.R.F. Department of Medical Oncology, St Bartholomew's Hospital, West Smithfield, London EC1A 7BE.

Stuart, 1975). More recently such tumours have been shown to express the phenotype of foetal thymic precursor cells (Stein *et al.*, 1976). Similarly, expression of the surface antigen associated with acute lymphoblastic leukaemia cells (ALL antigen; Roberts *et al.*, 1978) and the enzyme terminal deoxynucleotidyl transferase (TdT; McCaffrey *et al.*, 1975; Donlon *et al.*, 1977; Kung *et al.*, 1978) have both been shown to be markers of cellular immaturity rather than of malignancy. These observations strongly suggest that the surface phenotype of non-Hodgkin

TABLE 1.—*Published data relating phenotypic expression to lymphoid differentiation in non-Hodgkin lymphoma, leukaemia and normal subjects*

| Profile | Class of malignancy | Normal equivalent | References |
|---|---|---|---|
| $ALL^+$, $Ia^+$, $TdT^+$ | ALL/ML LB/DU | Lymphoid stem cell | Roberts *et al.*, 1978; Janossy *et al.*, 1977; Hoffbrand *et al.*, 1977. |
| $ALL^+$, $Ia^+$, $TdT^+$, $CyIgM^+$ | ALL | Pre-B cell | Vogler *et al.*, 1978; Pearl *et al.*, 1978. |
| $HTLA^+$, $TdT^+$, $E^-$ | ALL/ML LB/DU | Pre-thymic T cell | Kersey *et al.*, 1974; Greaves *et al.*, 1977. |
| $E^+$, $HTLA^+$, $TdT^+$ | ALL/ML LB(T)/DU | Thymic T cell | McCaffrey *et al.*, 1975; Greaves, 1978; Hann *et al.*, 1977 |
| $E^+$, $C3^+$, $TdT^+$ | Sternberg sarcoma/ALL ML LB(T)/DU | T precursor | Barrett *et al.*, 1977; Stein *et al.*, 1976; Kung *et al.*, 1978; Jaffe *et al.*, 1977. |
| $E^+IgM^+Fc^+C3^+(Ia^+)$ | Cells associated with CLL, ALL, HD, lymphoma | Functional T cell Subsets | Chapel & Ling, 1975; Dickler *et al.*, 1974; Bolhuis & Nooyen, 1977; Habeshaw *et al.*, 1976; Moretta *et al.*, 1977; Greaves *et al.*, 1978; Toben & Smith, 1977; McConnell & Hurd, 1976; Wernet & Wilms, 1977; Chiao *et al.*, 1974; Ferrarini *et al.*, 1975. |
| $SIg^+$ (Weak expression) | CLL/MLL/DWDL | Immature B cell | Pilcher & Knapp, 1977. |
| $SIg^+Fc^+IgM^+$ | CLL/MLL/DWDL | Immature B cell | Pilcher & Knapp, 1977. |
| $SIg^+Fc^+IgM^+C3^+$ | CLL/MLL/DWDL | ? Virgin B cell | This paper. |
| $SIg^+Fc^+C3^+$ | ? CLL/MLL/DWDL | ? Memory B cell/ mantle zone lymphocyte | Braylan *et al.*, 1977. This paper. |
| $SIg^+C3^+$ | CB/cc/F or D N or DPDL | Germinal follicle centre cell | Stein *et al.*, 1978; Jaffe *et al.*, 1974; Stein, 1976. |
| $SIg^+$ (capping) | NPDL, DPDL, cc/Sc, CB/cc/F | Medullary cord lymphocyte (pro-plasma cell) | Stuart & Habeshaw, 1976; Jaffe *et al.*, 1977. |
| $SIg^-$ (non-capping) | ML IB/DHL | "Plasmablast" pro-plasma cell | Habeshaw *et al.*, 1977; This paper |
| $SIg^+CyIg^+$ | ML IB/DH "Burkitt"/DU | ? Pro-plasma cell | This paper; Brunning *et al.*, 1977. |
| $CyIg^+$ | Plasmacytoma | Plasma cell | This paper; Harris, 1974. |
| Receptor-silent cell ($E^-$, $SIg^-$, $HTLA^-$, $ALL^-$, $Ia^-$, $Fc^-$, $C3^-$, $CyIg^-$) | ML IB/DH | Present in normal tissue | Belpomme *et al.*, 1977; Habeshaw & Stuart, 1975; This paper. |

*Histological abbreviations—Rappaport:* DH = diffuse histiocytic; DM(L+H) = diffuse mixed lymphocytic and histiocytic; DPDL = diffuse poorly differentiated lymphocytic; DU = diffuse undifferentiated; DWDL = diffuse well differentiated lymphocytic; NM(L+H) = nodular mixed lymphocytic and histiocytic; NPDL = nodular poorly differentiated lymphocytic; (A) = atypical.

*Kiel:* D = diffuse; F = follicular; F+D = follicular and diffuse; Lc = large cell; ML CB = malignant lymphoma centroblastic; ML CB/cc = malignant lymphoma centroblastic and centrocytic; ML CC = malignant lymphoma centrocytic; ML Hg Pl = malignant lymphoma high grade pleomorphic; ML Hg U = malignant lymphoma high grade unclassified; ML IB = malignant lymphoma immunoblastic; MLL = malignant lymphoma lymphocytic; ML LB = malignant lymphoma lymphoblastic; ML LB(B) = malignant lymphoma lymphoblastic of B type; ML LB(T) = malignant lymphoma lymphoblastic of T type; ML Lg U = malignant lymphoma low grade unclassified; ML Lp = malignant lymphoma lymphoplasmacytoid; Pb = plasmablastic; Sc = small cell.

lymphoma cells identifies cells which have undergone maturation arrest at various critical points in their normal differentiation and maturation sequence. The phenotypes of malignant lymphoma cells and their relationships to normal lymphoid cells of equivalent phenotype are shown in Table I and discussed in the references given.

The purpose of this study was threefold. Firstly, a panel of membrane and cytoplasmic markers was developed and standardized. The expression of surface immunoglobulin (SIg), the redistribution of SIg molecules on the cell surface, the expression of C3d, Fc$\gamma$ and Fc$\mu$ receptors and of cytoplasmic immunoglobulin (CyIg) are all probably related to the different stages of B-cell maturation and function, and their inclusion in a "receptor profile" of lymphoma is important. Secondly, the dominant cell populations in various normal and reactive lymphoid tissues were analysed with these markers. Thirdly, the characteristics of neoplastic populations from 157 cases of non-Hodgkin lymphoma were established, and correlated with the histopathological and cytological features of these tumours. The sequential analysis of serial samples from some of these patients suggested certain developmental links between the different subclasses of lymphoma and their normal equivalent B-cell subtypes.

### MATERIALS AND METHODS

Tissues from 157 patients with non-Hodgkin lymphoma were studied. These included 44 patients with CLL whose blood was studied. Single-cell suspensions were prepared from various lymphoid organs. Blood, marrow and spleen suspensions were separated on Ficoll–Triosil, and surface marking performed on the recovered white-cell layers. Touch preparations were made from the cut lymph node surface and cytocentrifuge preparations from cell suspensions. These were studied by cytology and cytochemistry. Trephine biopsy samples of marrow and paraffin-embedded preparations were studied by histology. Histological and cytological diagnoses were made independently of knowledge of the receptor profile. The determination of the receptor profile of single-cell suspensions was carried out as follows.

### *Preparation of rosettes*

*E rosettes.*—$10^6$ washed leucocytes were mixed with $40 \times 10^6$ washed sheep RBC in 500 $\mu$l of Medium 199, incubated for 10 min at 37°C, centrifuged for 5 min at 100 *g*, and incubated in the pellet for 2 h at 4°C. After gentle resuspension, 5 $\mu$l of 0·1% solution of acridine orange was added, and the preparation examined wet using a microscope with a tungsten–halogen light source and dichroic filtered (narrow-band blue/red) light. The stained nuclei fluoresce green, enabling the easy identification of rosetting cells. Nucleated intact cells binding more than 3 red cells were counted as rosettes.

*Fc rosettes.*—Rabbit anti-ox-RBC IgG was prepared from hyperimmune rabbit serum by $(NH_4)_2SO_4$ precipitation and DEAE–cellulose chromatography. Optimal titre for red-cell sensitization was determined as that titre giving the maximum level of Fc rosetting by normal blood mononuclear cells without agglutination. The numbers of rosettes formed with increasing amounts of antibody on the red cells shows a plateau at 20–35% rosettes with samples of normal blood mononuclear cells.

*IgM rosettes.*—Rabbit anti-ox-RBC IgM was prepared by the i.v. injection of rabbits with 2·5 mg/ml (wet wt) of ox erythrocyte stroma suspended in 5% gelatin solution (Kabat & Mayer, 1961). The first peak on G-200 Sephadex chromatography (IgM) was separated. IgM titres for red cell sensitization (IgM rosettes) were selected by titration of antibody dose against percentage rosettes using $IgM^+$ CLL cells. Kinetics of IgM rosetting were similar to Fc rosetting, although IgM rosettes are not formed in the presence of free human IgM. Red cells sensitized for 30 min at 37°C were used to prepare rosettes by the same method as for E rosettes.

*C3d rosettes.*—Ox RBC were coated with a titre of rabbit-anti-ox-RBC IgM antibody found to give optimal complement binding. Sensitized cells were then treated with human R3 reagent (Lachmann *et al.*, 1973) and washed twice. The receptor expressed is the C3d receptor, since such cells react with anti-human C3 antisera but are immuneadherence-negative. Rosetting was performed as for E rosettes.

*Quantitation of phagocytes*

Phagocytes were quantitated by mixing $10^6$ WBC in 500 $\mu$l Medium 199 with 10 $\mu$l of a 1% aqueous solution of Neutral Red dye (Gurr). After 15min incubation at 37°C the cells were washed once in Medium 199, resuspended in 10 $\mu$l and counted. Macrophages show coarse clumped granular uptake of the dye, myeloid cells a fine stippled uptake, and lymphoblasts single discrete granules. Dead cells show red nuclear staining.

*Surface immunoglobulin staining and acetate washing*

Non-specific binding of immune complexes or immunoglobulin to the cell surface *in vitro* or *in vivo* can lead to misinterpretation of SIg patterns detected by immunofluorescence. Following reports that SIg expression could be enhanced by washing in acetate buffer at low pH (Hutteroth *et al.*, 1972) the following technique has been found useful in determining SIg expression by lymphoma cells.

Acetate buffer, at pH 5·5 containing 8·5 g/l NaCl and 1·0 g/l $CaCl_2$ was prepared. 10 ml of buffer was added to 10–20 × $10^6$ cells and the suspension incubated for 10–15 min at 37°C. The cells were then washed once in buffer and once in Medium 199, and incubated for 1 h at 37°C. This procedure readily displaces non-specifically bound Ig from CLL B cells, and lymphoma B and T cells, without affecting expression of membrane-associated (synthesized) Ig.

*Surface Ig staining*

SIg staining was performed on acetate-buffer-washed cells. $10^6$ cells were incubated with FITC-conjugated antisera against $\gamma$, $\mu$, $\alpha$, $\kappa$ and $\lambda$ chains for 30 min at 4°C, washed twice, and $SIg^+$ cells counted using a Zeiss photomicroscope 111 with incident UV illumination and transmitted light for phase contrast. Commercial antisera (Meloy, Burroughs Wellcome, Dako, Behringwerke) were used after appropriate titration and specificity tests. All antisera were deaggregated by centrifugation at 110,000 *g* for 1 h before use.

*Distribution of SIg, capping and non-capping cells*

Direct staining for SIg allows observation of the movement of the SIg-anti-Ig complex on the cell surface. To demonstrate the variable kinetics of the capping reaction in B cells of different subclass, a sandwich technique was used. In this $10^6$ cells were stained with polyvalent rabbit anti-human $\gamma$, $\mu$, $\alpha$ heavy-chain antiserum at 4°C for 30 min. After being washed twice in ice-cold PBS, the cells were stained with FITC-coupled goat anti-rabbit serum (Meloy) or sheep anti-rabbit serum (Burroughs Wellcome) for 30 min at 4°C. After further washing in cold PBS, the cells were warmed at 37°C for 15–20 min to allow capping to occur, and examined immediately. This technique produces rapid and consistent capping by nearly all normal B cells. In some lymphomas the SIg remains localized as small dots or patches on the membrane (non-capping) or the cells show "slow capping" after 1–4 h incubation. In cases (*e.g.* CLL) where SIg staining is weak, it was not always possible to assess capping.

*Cytoplasmic Ig staining*

Cytocentrifuge preparations were fixed in cold absolute ethanol, brought to PBS, and stained with 100 $\mu$l of the appropriate dilutions of FITC-coupled antisera against $\gamma$, $\mu$, $\alpha$, $\delta$, $\kappa$ and $\lambda$ chains of immunoglobulin. After 2h washing in PBS, preparations were mounted in Uvinert Aqueous (Gurr) and examined for intracytoplasmic fluorescence.

*Special staining*

Where no E, Fc, C3, IgM or SIg was detected on the cells, they were examined with anti-ALL, anti-Ia and anti-HTLA sera kindly provided by Drs M. Greaves, M. Roberts and G. Janossy (ICRF Laboratories).

*Classification of lymphomas by receptor profile*

The proportions of different cell types in the lesion were determined. T cells were defined by E rosetting or HTLA positivity, B cells by the presence of acetate-wash-resistant SIg. Macrophages were defined by Neutral Red ingestion and expression of Fc receptor, and plasma cells had CyIg. Certain cells lacked all markers (receptor-silent cells). The samples were classified according to the major cell type present.

*T-cell-predominant tumours.*—These could be divided into those which have (a) <10% of other cell types, (b) >10% of polyclonal B cells ("T-predominant polyclonal B") and

(c) >10% of monoclonal B cells ("T-predominant monoclonal B"). In the T-cell-predominant group (a), expression of Fc, C3 or IgM receptors was accounted positive if expressed on more than 25% of the cells.

*B-cell-predominant tumours*.—These were classified by the type of SIg expressed, and by the subsidiary cell markers Fc, IgM and C3. In most cases Fc and C3 receptors were clearly expressed on the majority (B-cell) population. In samples containing substantial numbers of T cells it was not certain that Fc and C3 receptor expression was limited to the B-cell fraction. In a few cases, expression of Fc and/or C3 receptor by the B cell was confirmed by the analysis of T-cell-depleted populations. This was achieved by the elimination of E rosetting cells on Ficoll–Triosil. Fc, C3, IgM receptors were counted as positive if expressed by more than 25% of viable cells in the sample.

In B-cell-predominant lymphomas and CLL the following B-cell profiles were obtained:

(1) Capping $SIg^+$

(2) Non-capping $SIg^+$

(3) Weak $SIg^+$

(4) $SIg^+Fc^+IgM^+$

(5) $SIg^+Fc^+$

(6) $SIg^+Fc^+IgM^+C3^+$

(7) $SIg^+Fc^+C3^+$

(8) $SIg^+$(Non-capping)$C3^+$

(9) $SIg^+C3^+$

(10) $SIg^+CyIg^+$

(11) $SIg^-CyIg^+$

*Surface immunoglobulin*

The expression of $\kappa$ and $\lambda$ light chains was used to assess the monoclonality of SIg. $\kappa$-Chain monoclonality was arbitrarily defined as a $\kappa/\lambda$ chain ratio of 10 or greater. $\lambda$-Chain monoclonality was defined as a $\lambda/\kappa$ chain ratio of 5 or greater. Where 2 heavy chains were expressed on the majority of $SIg^+$ cells in a monoclonal tumour, the lesion was classified under both heavy-chain classes.

### RESULTS—I

*Normal tissues*

*Classification of normal tissues by surface marking*.—Amongst the 37 normal and reactive lymph nodes studied T cells were the predominant population in 12 nodes, T cells and B cells were present in roughly equal proportions in 22 nodes and B cells were predominant in 3 nodes. Nodes contained mixtures of B cells of different phenotype. The B-cell population in normal or reactive nodes expressed C3 receptors only (13 cases), C3 and Fc receptors (5 cases) or SIg only (19 cases). Numbers of $CyIg^+$ cells were variable, but generally <10%. In 24 tonsil samples, B cells were the predominant population in 18 cases, and B and T cells present in equal numbers in 6 cases. Plasma cells formed 2–11% of the tonsil cell population. Almost all B cells in tonsil expressed C3d receptors only. After elimination of T cells, 5–23% of tonsil cells were $SIg^-$ receptor-silent "null cells". Some of these cells expressed Ia antigen, but were $HTLA^-$ and $CyIg^-$. In the 10 spleen samples studied, T cells were the predominant population in 4, and T and B cells present in equal proportion in 6. Spleen B cells most commonly expressed both Fc and C3 receptors, 10–24% of the spleen cells were receptor-silent and 3–15% were identified as mononuclear phagocytes. In all these samples, the B cells seen expressed either $\kappa$ or $\lambda$ light chains, and monoclonal B-cell populations were never seen.

### RESULTS—II

*Peripheral blood in CLL (Table II)*

In all but 2 of the 44 cases examined, monoclonal SIg could be detected after acetate washing and incubation of the cells. In 2 cases, removal of polyclonal SIg was followed by failure to detect any SIg on the cells. In all samples studied, T cells formed less than 10% of the circulating population. SIg expression was invariably weak, and single-chain only (most commonly $\mu$ or $\kappa$) in 26 cases. The surface phenotype of the cases of CLL seen is given in Table II. The most consistent feature was the high incidence of IgM receptor expression by CLL B cells in peripheral blood (16/44). CLL B cells expressing C3d receptor only were seen in

TABLE II.—*Surface phenotype in 44 cases of chronic lymphocytic leukaemia (CLL)*

| Profile | No. of cases |
|---|---|
| $SIg^+Fc^-C3^-IgM^-$ | 16 |
| $SIg^+Fc^+C3^-IgM^+$ | 4 |
| $SIg^+Fc^+C3^+IgM^+$ | 12 |
| $SIg^+Fc^+C3^+IgM^-$ | 4 |
| $SIg^+Fc^-C3^+IgM^-$ | 6 |
| $SIg^-Fc^-C3^-IgM^-$ | 2 |

Key: $SIg^+$=All B cells exhibit monoclonal SIg resistant to acetate washing. $Fc^+$, $C3^+$, $IgM^+$=Expression of receptor on more than 25% of B cells.

6/44 cases. Of the cases expressing light-chain determinants (35/44), far more expressed $\kappa$ (29) than $\lambda$ (6).

### RESULTS—III

### *Surface phenotype in diffuse undifferentiated lymphoma* (DU)

The phenotype of 18 biopsy specimens from 16 patients with DU are shown in Table III. In 6 patients the phenotype corresponded to that of T-cell malignancy. One patient (Bre.) had the phenotype of $E^+$ and $C3d^+$ T cells. In 2 patients (Leg., Mad.(2)) the cells expressed HTLA, but were $E^-$. In 5 patients (Par., Hub., Edm., Bre., Hug.) the cells showed the cytological features of T lymphoblasts (convoluted nuclei, acid-phosphatase-positive). The cells of patients Mad. and Leg. did not show these features. Patients Par., Edm., Hug. were leukaemic at the first biopsy (WBC$>5\pm10^9$/l) and showed X-ray evidence of a mediastinal mass. In Par. the lesion progressed to involve the CSF, and the CSF cells were predominantly of T-cell type. In Patient Mad. the initial diagnosis (histology and cytology) was that of high-grade pleomorphic lymphoma, and the phenotype was of $E^+$ cells. The second biopsy showed lymphoblastic lymphoma without the characteristic cytological features of T lymphoblasts, and the cells were $E^-$ but $HTLA^+$.

Four patients (Fel., Guy., Shep., Mas.) had lesions composed of undifferentiated lymphoblasts, and all were leukaemic on biopsy. In 3 cases the cells were $ALL^+$ and $Ia^+$, and in the 4th case insufficient cells were obtained for testing. The lesions in these patients correspond to ALL with nodal involvement. Five patients (Ber., Fay., Bry., Bal., Gre.) had B-cell neoplasms. In 3 cases (Fay., Bal., Gre.) the lymphoblasts showed Burkitt-lymphoma-like features, allowing a cytological diagnosis of B lymphoblastic lymphoma. In 4 patients (Ber., Tay., Bry., Bal.) the malignant cells failed to cap their SIg. Patient Fay. was leukaemic at diagnosis, and it is of interest that the marrow lymphoblasts, but not the blood lymphoblasts, showed the presence of CyIgM. Patient Gre. presented with an ulcerating scalp tumour in which a minority of the lymphoblasts expressed CyIgM.

### *Surface phenotype in diffuse histiocytic lymphoma* (DH) (*Table IV*)

Of the 17 cases of DH in this series, 12 had lesions containing a monoclonal B-cell component. In 6 cases the B cells failed to cap SIg, and in 3 patients capping B cells were the major population. One patient (God.) had polyclonal capping B cells as the major component of his tumour. Two patients (Smi., Mor.) had lesions showing T-cell predominance associated with monoclonal B cells, and one patient (Lee.) had CyIg-containing B cells as the major component of the lesion. Patients Smi. (1, 2), Kos. (3), Has. (2) and Lov. (1, 2, 3) had repeat biopsies. Two patients had T-cell-predominant tumours (Ham., Lov.). Lov. had a T-cell-predominant tumour which became $E^-$ in a subsequent biopsy (not tested with anti-HTLA). Ham. had an immunoblastic lymphoma of T-cell type. One patient (Tun.) had a true receptor-silent tumour ($E^-$ $SIg^-$ $ALL^-$ $HTLA^-$). Two patients (Byi., Fin.) were judged to have malignant histiocytosis on cytology and cytochemistry. In both biopsy specimens large numbers of functional phagocytes were present. In Patient Fin. these cells were accompanied by a B-cell population expressing $\kappa$ chain only. Large receptor-

TABLE III.—*Surface markers expressed in malignant lymphoma of diffuse undifferentiated (DU) histological type*

| Patient | Age | Sex | Source | Kiel | E | Fc | C3 | IgM | Phag | Caps | Non-caps | CyIg | Ig class | Profile |
|---|---|---|---|---|---|---|---|---|---|---|---|---|---|---|
| Par. | 3 | F | Node | ML LB(T) | 53 | 16 | 26 | 5 | 2 | 15 | 0 | — | Pcl* | T predominant |
| Hub. | 32 | F | Node | ML LB(T) | 79 | 15 | 17 | NA | 15 | 11 | 0 | — | Pcl | T predominant |
| Edm. | 21 | M | Node | ML LB(T) | 90 | 0 | 0 | NA | <1 | 2 | 0 | — | — | T predominant |
| Bre (1) | 4 | M | Node | ML LB(T) | 70 | 16 | 26 | 24 | 2 | 15 | 0 | — | Pcl | $E^+C3^+$ |
| Bre (2) | 5 | M | Eff. | ML LB(T) | 73 | 2 | 64 | 5 | 2 | 3 | 0 | — | — | $E^+C3^+$ |
| Hug. | 6 | M | Node | ML LB(T) | 72 | <1 | 3 | <1 | <1 | 5 | 0 | — | — | T predominant |
| Mad | 67 | M | Node | ML Hg Pl | 89 | 0 | 2 | 0 | 1 | 5 | 0 | — | — | T predominant |
| Mad (2) | 67 | M | Node | ML LB | 24 | 3 | 3 | 2 | 2 | 10 | 0 | — | Pcl | ($HTLA^+$) |
| Leg. | 11 | M | Eff. | ML LB | 43 | 1 | 10 | 2 | 1 | 0 | 0 | — | — | ($HTLA^+$) |
| Fel. | 23 | M | Node | ML LB | 6 | 0 | 0 | NA | <1 | 4 | 0 | — | — | $ALL^+$ $Ia^+$ |
| Guy. | 28 | F | Node | ML LB | 4 | 3 | 0 | 0 | 2 | 2 | 0 | — | — | $ALL^+Ia^+$ |
| Shep. | 3 | M | Node | ML LB | 2 | 7 | 4 | 2 | 2 | 0 | 0 | — | — | $ALL^+Ia^+$ |
| Mas. | 4 | M | Node | ML LB | 9 | 3 | 3 | 0 | 0 | 1 | 0 | — | — | Not tested |
| Ber. | 55 | F | Eff. | ML LB | 1 | 14 | 8 | 0 | 6 | 20 | 50 | — | $\gamma\mu\kappa$ | Non-capping B |
| Fay. | 6 | M | Marrow | ML LB(B) | 6 | 8 | 13 | 18 | 0 | 40 | 55 | $\mu\kappa^+$ | $\mu\kappa$ | Non-capping B $CyIg^+$ |
| Bry. | 65 | M | Node | ML LB | 10 | 15 | 4 | 1 | 5 | 20 | 65 | — | $\mu\lambda$ | Non-capping B |
| Bal. | 75 | F | Eff. | ML LB(B) | 4 | 0 | 0 | NA | 0 | 3 | 58 | — | $\mu\kappa$ | Non-capping B |
| Gre. | 2 | M | Scalp nodule | ML LB(B) | 4 | 0 | 4 | 2 | <1 | 69 | 7 | $\mu\kappa^+$ | $\gamma\mu\kappa$ | Capping $SIg^+$ |

*Note:* Patients Edm., Fel., Guy., and Shep. were leukaemic at time of biopsy—all were diagnosed as ALL.

* Pcl=polyclonal SIg; NA=data not available.

TABLE IV.—*Surface markers expressed in cases of diffuse histiocytic (DH) malignant lymphoma*

| Patient | Age | Sex | Source | Kiel | E | Fc | C3 | IgM | Phag | Caps | Non-caps | CyIg | SIg class | Profile |
|---|---|---|---|---|---|---|---|---|---|---|---|---|---|---|
| Bur. | 59 | M | Node | ML IB | 6 | 14 | 17 | 0 | 10 | 15 | 60 | — | $\gamma\kappa$ | SIg+ Non-capping |
| Aga. | 22 | F | Node | ML IB | 5 | 0 | 3 | NA | 0 | 22 | 49 | — | $\mu\lambda$ | SIg+ Non-capping |
| Woo. | 46 | F | Node | ML IB(PB) | 11 | 15 | 20 | 0 | 4 | 2 | 81 | — | $\gamma\lambda$ | SIg+ Non-capping |
| Smi (2) | 52 | M | PNS | ML CB+IB | 10 | 3 | 9 | 14 | <1 | 8 | 71 | — | $\gamma\mu\kappa$ | SIg+ Non-capping |
| Kee. | 54 | F | Node | ML IB(PB) | 14 | 3 | 3 | 0 | 1 | 15 | 78 | — | $\mu\lambda$ | SIg+ Non-capping |
| Has (2) | 71 | F | Node | ML IB | 1 | 1 | 20 | 4 | 1 | 38 | 58 | — | $\mu\kappa$ | SIg+ Non-capping |
| Kos (3) | 53 | M | Node | ML IB | 8 | 2 | 2 | 0 | 2 | 10 | 80 | — | $\mu\kappa$ | SIg+ Non-capping |
| Sau. | 8 | F | Tonsil | ML IB | 39 | 15 | NA | NA | 0 | 12 | 43 | — | $\gamma\mu\kappa$ | SIg+ Non-capping |
| Hug. | 34 | M | Node | CB/cc/D IB | 32 | 9 | NA | NA | 0 | 5 | 55 | — | $\mu\kappa$ | SIg+ Non-capping |
| Far. | 58 | M | Node | ML IB | 28 | 2 | 2 | 2 | 1 | 73 | 2 | — | $\gamma\mu\kappa$ | SIg+ |
| God. | 20 | M | Node | ML IB | 18 | 5 | 12 | 17 | 2 | 56 | 0 | — | Pcl | SIg+ |
| Eps. | 70 | F | Node | ML IB | 17 | 28 | 24 | 10 | 3 | 71 | 0 | — | $\gamma\lambda$ | SIg+Fc+C3+ |
| Lee. | 69 | F | Node | ML IB | 23 | 6 | 6 | 1 | 6 | 25 | 0 | 40 $\kappa$ | $\kappa$ | SIg+CyIg+ |
| Smi (1) | 52 | M | Node | ML CB | 50 | 5 | 7 | NA | 0 | 40 | 0 | — | $\mu\kappa$ | T pred. Mcl B |
| Mor. | 48 | M | Node | ML CB | 66 | NA | 14 | 0 | 10 | 15 | 0 | — | $\mu\kappa$ | T pred. Mcl B |
| Ham. | 32 | F | Node | ML IB | 85 | 0 | 0 | NA | 4 | 1 | 0 | — | — | T predominant |
| Lov (1) | 51 | F | Node | ML CC/Lc | 74 | 1 | NA | NA | 1 | 32 | 3 | — | Pcl | T pred. Pcl B |
| Lov. (2) | 52 | F | Node | ML CC/Lc | 60 | 0 | 0 | 0 | <1 | 7 | 0 | — | — | T predominant |
| Lov. (3) | 52 | F | Node | ML CC/Lc | 5 | 2 | 7 | 0 | 2 | 5 | 0 | — | — | RS |
| Tun. | 78 | F | Node | ML IB | 1 | 2 | 3 | 0 | 0 | 0 | 0 | — | — | RS |
| Byi. | 74 | M | Node | M. Hist. | 27 | 35 | 0 | NA | 35 | 0 | 0 | — | — | M cell |
| Fin. | 6 | F | Node | M. Hist. | 4 | 14 | 23 | 0 | 20 | 35 | 0 | — | $\kappa$ | M+Mcl B |

*Note:* Mcl=monoclonal; Pcl=polyclonal; M cell=macrophage; NA=data not available.

TABLE V.—*Surface markers expressed in diffuse poorly differentiated lymphocytic lymphoma (DPDL) and diffuse mixed lymphocytic and histiocytic lymphoma (DM (L+H))*

| Patient | Age | Sex | Source | Rappaport | Kiel | E | Fc | C3 | IgM | Phag | Caps | Non-caps | CyIg | Ig class | Profile |
|---|---|---|---|---|---|---|---|---|---|---|---|---|---|---|---|
| Mur. | 45 | M | Spleen | DPDL | ML/cc/Lc | 5 | 0 | 5 | NA | 0 | 0 | 70 | — | $\mu\lambda$ | Non-capping B |
| Ver. | 51 | M | Spleen | DPDL | ML/cc/Pl | 15 | 1 | 10 | 1 | 9 | 10 | 65 | — | $\mu\kappa$ | Non-capping B |
| Dat. | 54 | M | Node | DPDL | ML/cc/Lc | 3 | 8 | 3 | NA | 1 | 0 | 66 | — | $\mu\lambda$ | Non-capping B |
| McH. | 52 | M | Node | DPDL | ML/cc/Lc | 5 | 17 | 4 | NA | 0 | 3 | 75 | — | $\mu\lambda$ | Non-capping B |
| Bai. | 46 | F | Node | DPDL | ML/cc/Sc | 6 | 15 | 50 | 0 | 1 | 19 | 56 | — | $\mu\lambda$ | SIg+C3+Nc |
| Car. | 64 | F | Node | DPDL | ML/cc/Sc | 23 | 0 | 28 | 0 | 0 | 0 | 83 | — | $\mu\kappa$ | SIg+C3+Nc |
| Rob. | 60 | M | Node | DPDL | ML/cc/Sc | 11 | 1 | 34 | 0 | 0 | 3 | 85 | — | $\gamma\mu\lambda$ | SIg+C3+Nc |
| Koz. (1) | 53 | M | Node | DPDL(A) | ML Lpc | 47 | 4 | 56 | NA | NA | 50 | 0 | — | $\mu\kappa$ | SIg+C3+ |
| Col. (1) | 48 | M | Node | DPDL | CB/cc/D | 32 | 10 | 33 | 4 | 0 | 70 | 0 | — | $\gamma\mu\kappa$ | SIg+C3+ |
| Chal. | 64 | M | Node | DPDL | ML/cc/Lc | 15 | 17 | 40 | 9 | 2 | 63 | 0 | — | $\gamma\mu\lambda$ | SIg+C3+ |
| Bau. | 64 | F | Node | DPDL | ML/cc/Sc | 6 | 20 | 65 | 0 | 1 | 70 | 0 | — | $\gamma\lambda$ | SIg+C3+ |
| Wil. | 60 | F | Node | DPDL | ML/Lg U | 36 | 8 | 25 | 1 | 36 | 15 | — | — | $\gamma\mu\lambda$ | SIg+C3+ |
| Ren. | 58 | M | Node | DPDL | CB/cc/D | 12 | 0 | 75 | 5 | 1 | 80 | 0 | — | $\mu\lambda$ | SIg+C3+ |
| Sea. | 68 | M | Node | DPDL | CB/cc/D | 16 | 4 | 64 | 4 | $<1$ | 64 | 20 | — | $\alpha\kappa$ | SIg+C3+ |
| Hil. | 56 | M | Node | DPDL | CB/cc/D | 12 | 1 | 26 | 2 | $<1$ | 67 | 0 | — | $\gamma\mu\kappa$ | SIg+C3+ |
| Mat. | 58 | F | Node | DPDL | ML/Lg U | 4 | 1 | 35 | $<1$ | 7 | 35 | 0 | 10 $\gamma\kappa$ | $\gamma\kappa$ | SIg+C3+CyIg+ |
| Col. | 54 | F | Marrow | DPDL(A) | ML/Lg U | 20 | 0 | 0 | 0 | 0 | 18 | 0 | 25 $\mu\kappa$ | $\mu\kappa$ | SIg+CyIg+ |
| Pet (1) | 63 | M | Node | DPDL | ML/cc/Sc | 13 | 8 | 8 | 0 | 0 | 82 | 0 | — | $\mu\lambda$ | SIg+ |
| Pet (2) | 63 | M | Node | DPDL | ML/cc/Sc | 3 | 6 | 8 | 0 | 0 | 78 | 2 | — | $\mu\lambda$ | SIg+ |
| Leyf. | 56 | F | Node | DPDL | ML/cc/Sc | 2 | 1 | 6 | 0 | 5 | 80 | 0 | — | $\mu\kappa$ | SIg+ |
| Rob. | 61 | F | Node | DPDL | CB/cc/D | 17 | 20 | 10 | 12 | 2 | 51 | 5 | — | Pcl | SIg+ |
| Bri. (2) | 65 | F | Node | DM(L+H) | CB/cc/D | 15 | 4 | 3 | 3 | 2 | 52 | 0 | — | Pcl | SIg+ |
| Nab. | 55 | M | Node | DPDL | ML Lpc | 23 | 1 | 24 | 1 | 2 | 44 | 0 | — | $\gamma$ | SIg+ |
| Mat. | 75 | M | Node | DPDL | ML Lpc | 55 | NA | NA | NA | $<1$ | 40 | 0 | — | $\gamma\mu\lambda$ | T pred. Mcl B |
| Koz. (2) | 54 | M | Node | DPDL(A) | ML/LgU | 60 | 17 | 18 | 0 | 23 | 19 | 4 | — | $\kappa$ | T pred. Mcl B |
| Godr. | 20 | F | Node | DPDL | ML/HgU | 60 | 16 | 36 | NA | 5 | 17 | 0 | — | Pcl | T pred. Pcl B |
| Gri. | 49 | M | Eff. | DPDL | ML/cc/Sc | 63 | $<1$ | 2 | 0 | $<1$ | 0 | 5 | 10 $\gamma\kappa$ | Pcl | T pred. Pcl B |

silent cell populations were also present in these cases.

Comparing the Kiel classification with the surface markers in this group, the diagnosis of "immunoblastic malignant lymphoma" (ML IB) corresponded closely with B-cell predominant tumours, the exception being patient Tun. who had a receptor-silent neoplasm. In 2 patients, Smi. (2), Hug., the diagnosis indicated immunoblastic transformation in a centroblastic and centrocytic tumour. Both T-cell-predominant monoclonal B-cell tumours were diagnosed as "malignant lymphoma centroblastic" in the Kiel classification. The cases of T-cell malignancy were classified as a "centrocytic tumour of large cell type", and as ML IB.

The evidence from this series shows that the majority of DH lymphomas are of B-cell derivation, and are composed of large cells which are slow to cap SIg anti-immunoglobulin complex. T-cell lymphomas are rare in this histological class. DH lymphomas which lack surface Ig, Fc, C3d and E receptors may contain CyIg, or may be receptor silent. True histiocytic malignancies are rare.

*Surface phenotype in diffuse poorly differentiated lymphocytic lymphoma (DPDL) (Table V)*

Twenty-four patients in this group contributed 27 biopsy specimens of involved tissue. In all but 4 patients the lesion examined was B-cell predominant. In the remaining 4 biopsy specimens, 2 showed T-cell predominance with monoclonal B cells, and 2 T-cell predominance with polyclonal B cells. No receptor-silent tumours occurred in this group. Four patients had non-capping B-cell tumours, all classified as centrocytic tumours of large or pleomorphic cell type (Kiel). Three patients had non-capping tumours expressing C3d receptor, all classified as centrocytic tumours of small cell type (Kiel). Nine patients had capping tumours expressing C3d receptors, and one of these (Mat.) showed monoclonal IgG in the cytoplasm of 10% of the tumour cells. Six biopsy specimens showed a B-cell-predominant pattern with the phenotype $SIg^+$, and 2 of these were polyclonal. Five patients had low-grade tumours which could not be definitively assigned to any single class in the Kiel classification, although the phenotype in two of these cases (Wil., Mat.) was that of the follicular tumour ($SIg^+$ $C3d^+$: Kiel equivalent centroblastic/centrocytic/diffuse).

*Surface phenotype in nodular poorly differentiated lymphocytic lymphoma (NPDL) (Table VI)*

Thirty-two patients in this class provided 47 profiles. The majority of patients (27/32) presented with tumours in which B cells were the predominant population, or with T-cell-predominant tumours containing monoclonal B cells. Non-capping B-cell tumours presented in 5 patients. In Gig. (3) the tumour cells capped slowly, rather than failing to cap at all. In 12 patients, the first lesion in the series showed a predominant population of B cells with $SIg^+C3d^+$ phenotype. This was the most frequent profile in tumours of this class. Seven patients had lesions in which capping $SIg^+$ cells were the major population. Four patients had T-cell-predominant lesions on presentation, and these patients all eventually showed B-cell monoclonality, in contrast to some of the T-cell-predominant tumours of DPDL, DH, or DU classes. No true nodular T-cell tumour has been seen in this series. Two tumours (Ayl. (1), Tay) showed the unusual profile of $SIg^+Fc^+C3^+$ B-cell predominance, and 2 tumours were receptor-silent (Eve., Spe.). Only one of the nodular tumours (Ted. (3a)) showed CyIg in more than 10% of cells. Correlating phenotype with histological classification, most of the lesions expressing only surface immunoglobulin ($SIg^+$ capping) showed sclerosis of the node on histological examination. All the lesions classified as "nodular poorly differentiated", or as "nodular mixed", were classified as "centroblastic centrocytic follicu-

TABLE VI.—*Surface markers expressed in nodular poorly differentiated lymphoma (NPDL) and nodular mixed lymphocytic and histiocytic lymphoma (NM (LHL))*

| Patient | Age | Sex | Source | Rappaport | Kiel | E | Fc | C3 | IgM | Phag | Caps | Non-caps | CyIg | Ig class | Profile |
|---|---|---|---|---|---|---|---|---|---|---|---|---|---|---|---|
| Cle. | 65 | M | Node | NPDL | CB/cc/F | 29 | 17 | 15 | 0 | 1 | 27 | 53 | — | μλ | Non-capping B |
| McK. | 61 | M | Node | NPDL | CB/cc/F | 11 | 1 | <1 | <1 | 5 | 0 | 79 | — | μκ | Non-capping B |
| Bar. | 74 | F | Node | NPDL | CB/cc/F | 10 | 10 | 11 | 0 | <1 | 5 | 55 | — | νδ | Non-capping B |
| Tay. | 34 | M | Node | NPDL | CB/cc/F | 7 | NA | NA | NA | <1 | 0 | 75 | — | μλ | Non-capping B |
| Gig. (3) | 29 | M | Node | NPDL | CB/cc/F | 10 | <1 | <1 | 0 | <1 | 0 | 80 | — | γλ | Slow capping B |
| Wil. (1) | 36 | M | Node | NPDL | CB/cc/F+Sc | 38 | 4 | 56 | NA | <1 | 50 | 6 | — | γκ | $SIg^+C3^+$ |
| Eva. (1) | 64 | F | Node | NPDL | CB/cc/F | 30 | 3 | 52 | 0 | 0 | 53 | 0 | — | γκ | $SIg^+C3^+$ |
| Has (1) | 71 | F | Node | NPDL | CB/cc/F | 35 | 0 | 45 | 0 | 0 | 40 | 24 | — | μκ | $SIg^+C3^+$ |
| Dep. (1) | 44 | F | Node | NPDL | CB/cc/F | 21 | 4 | 40 | 0 | 0 | 78 | 0 | — | μκ | $SIg^+C3^+$ |
| Dep. (2) | 44 | F | Spleen | NPDL | CB/cc/F | 15 | 2 | 30 | 0 | 2 | 75 | 0 | — | μκ | $SIg^+C3^+$ |
| Bro. (1) | 54 | M | Node | NPDL | CB/cc/F | 20 | 2 | 38 | 3 | 4 | 81 | 0 | — | μλ | $SIg^+C3^+$ |
| Bro. (2) | 54 | M | Spleen | NPDL | CB/cc/F | 31 | 8 | 35 | 0 | 4 | 60 | 0 | — | μλ | $SIg^+C3^+$ |
| Sul. (1) | 31 | M | Node | NPDL | CB/cc/F | 14 | 7 | 29 | 0 | 0 | 67 | 0 | — | Pcl | $SIg^+C3^+$ |
| Rum. | 59 | F | Node | NPDL | CB/cc/F | 0 | 0 | 42 | 0 | 1 | 76 | 4 | — | μλ | $SIg^+C3^+$ |
| Whi. | 3 | F | Node | NPDL | CB/cc/F | 16 | 15 | 48 | 0 | 3 | 67 | 0 | — | Pcl | $SIg^+C3^+$ |
| Cha. (1) | 55 | M | Node | NPDL | CB/cc/F | 5 | 14 | 60 | 2 | 2 | 75 | 5 | — | μλ | $SIg^+C3^+$ |
| Cha. (2) | 56 | M | Node | N+DPDL | CB/cc/F+D | 3 | 3 | 90 | 5 | 1 | 75 | 15 | — | μλ | $SIg^+C3^+$ |
| Gig. (2) | 28 | M | Node | NPDL | CB/cc/F | 20 | 6 | 36 | 9 | 2 | 58 | 6 | — | γμλ | $SIg^+C3^+$ |
| Bre. (1) | 74 | F | Node | NPDL | CB/cc/F | 40 | 0 | 50 | NA | NA | 50 | 2 | — | Pcl | $SIg^+C3^+$ |
| Bre (2) | 75 | F | Node | NPDL | CB/cc/F | 42 | 0 | 35 | 0 | 8 | 45 | 0 | — | Pcl | $SIg^+C3^+$ |
| Mer. | 61 | M | Node | N+DPDL | CB/cc/F+D+Sc | 18 | 1 | 36 | 1 | 3 | 58 | 0 | — | γλ | $SIg^+C3^+$ |
| Ayl. (2) | 52 | F | Node | NPDL | CB/cc/F+Sc | 12 | 2 | 26 | 1 | <1 | 52 | 8 | — | Pcl | $SIg^+C3^+$ |
| Bla. | 57 | F | Node | NPDL | CB/cc/F | 2 | <1 | 27 | <1 | <1 | 45 | 10 | — | μκ | $SIg^+C3^+$ |
| Bri (1) | 64 | F | Node | N−DPDL | CB/cc/F+D | 30 | 1 | 44 | NA | 1 | 44 | 0 | — | Pcl | $SIg^+C3^+$ |
| Sul. (2) | 31 | M | Node | NPDL | CB/cc/F | 20 | 5 | 38 | 0 | 15 | 42 | 6 | — | Pcl | $SIg^+C3^+$ |
| Eva. (2) | 64 | F | Node | NPDL | CB/cc/F | 70 | 2 | 24 | 4 | 0 | 26 | 2 | — | μκ | T pred. Mcl B |
| Kri. | 27 | F | Node | NM(L+H) | CB/cc/F | 46 | 3 | 22 | <1 | <1 | 35 | 0 | — | κ | T pred. Mcl B |
| Wat. | 34 | M | Node | NPDL | CB/cc/F | 56 | 1 | 25 | <1 | 5 | 20 | 8 | — | γκ | T pred. Mcl B |
| Gig. (1) | 27 | M | Node | NPDL | CB/cc/F | 73 | 0 | NA | NA | NA | 37 | 0 | — | Pcl | T pred. Pcl B |
| Cou. (1) | 39 | F | Node | NM(L+H) | CB/cc/F | 64 | NA | NA | NA | 6 | 4 | 0 | — | — | T predominant |
| Cou. (2) | 39 | F | Node | N+DPDL | CB/cc/F+CB/D | 62 | 14 | 18 | NA | 4 | 25 | 0 | — | μκ | T pred. Mcl B |
| Ayl. (1) | 52 | F | Node | NM(L+H) | CB/cc/F | 20 | 40 | 45 | 0 | 10 | 68 | 0 | — | Pcl | $SIg^+Fc^+C3^+$ |
| Fai. | 34 | M | Node | NPDL | CB/cc/F | 7 | 5 | 6 | NA | <1 | 48 | 17 | — | μκ | $SIg^+$capping |
| Thor. | 67 | M | Node | NPDL | CB/cc/F Schl | 11 | 1 | 17 | 0 | 1 | 64 | 9 | — | μκ | $SIg^+$ capping |
| Mar. | 40 | M | Node | N+DPDL | CB/cc/F+D | 15 | 1 | 10 | 1 | 0 | 40 | 20 | — | μλ | $SIg^+$ capping |
| Wil. (2) | 36 | M | Node | NPDL | CB/cc/F Scl | 29 | 3 | 8 | 9 | 5 | 36 | 0 | — | γκ | $SIg^+$ capping |
| Wil. (3) | 36 | M | Node | NPDL | CB/cc/F Scl | 15 | 5 | 2 | 4 | 4 | 56 | 0 | — | Pcl | $SIg^+$ capping |
| Mer. | 61 | M | Node | NPDL | CB/cc/F Scl | 15 | 1 | 8 | 1 | 1 | 56 | 0 | — | κ | $SIg^+$ capping |
| Whi. | 52 | F | Node | NPDL | CB/cc/F | 19 | 2 | 9 | 0 | 4 | 50 | 5 | — | μκ | $SIg^+$ capping |
| Hug. | 32 | M | Node | NPDL | CB/cc/F | 32 | 5 | 0 | NA | NA | 70 | 0 | — | μκ | $SIg^+$ capping |
| Sus. | 31 | M | Node | NPDL | CB/cc/F Scl | 25 | 3 | 0 | 1 | 1 | 65 | 0 | — | μλ | $SIg^+$ capping |
| Eve. | 60 | M | Node | NPDL | CB/cc/F | 22 | 10 | 10 | 0 | 11 | 0 | 0 | — | — | Receptor-silent |
| Spe. | 51 | M | Node | NPDL | CB/cc/F Scl | 12 | 0 | 8 | 0 | 5 | 0 | 0 | — | — | Receptor-silent |
| Ted. (1) | 32 | F | Node | NPDL | CB/cc/F | 22 | 1 | 3 | 0 | 5 | 38 | 40 | — | μλ | $SIg^+$ non-capping |
| Ted (2) | 32 | F | Node | NPDL | CB/cc/→CB | 10 | 3 | 14 | 1 | 0 | 62 | 9 | — | μλ | $SIg^+$ capping |
| Ted. (3) | 32 | F | Node | NPDL | CB/cc/F→ CB | 20 | 2 | 3 | 0 | <1 | 70 | 15 | — | μλ | $SIg^+$ capping |
| Ted. (3a) | 32 | F | Node | NPDL | CB/cc/F | 40 | 1 | 8 | 0 | 0 | 40 | 25 | 12 γλ | μλ | $SIg^+$ capping, $CyIg^+$ |

TABLE VII.—*Surface markers expressed in diffuse well differentiated lymphocytic lymphoma (DWDL)*

| Patient | Age | Sex | Source | Kiel | E | Fc | C3 | IgM | Phag | Caps | Non-caps | CyIg | Ig class | Profile |
|---|---|---|---|---|---|---|---|---|---|---|---|---|---|---|
| Tuc. (1) | 45 | F | Node | MLL | 10 | 45 | 9 | 20 | 0 | 90 | | — | $\mu$ | SIg$^+$Fc$^+$ |
| Bas. | 70 | M | Node | MLL | 14 | 30 | 6 | NA | 0 | 65 | | — | $\lambda$ | SIg$^+$Fc$^+$ |
| Thac. | 62 | M | Node | ML Lp | 32 | 36 | 36 | NA | 5 | 60 | 2 | — | $\mu\kappa$ | SIg$^+$Fc$^+$C3$^+$ |
| Tuc. (3) | 46 | F | Spleen | MLL | 5 | 35 | 39 | 25 | 0 | 90 | | — | $\mu\kappa$ | SIg$^+$Fc$^+$C3$^+$IgM$^+$ |
| Mar. | 73 | M | Node | MLL | 6 | 32 | 52 | <1 | <1 | 30 | | — | $\kappa$ | SIg$^+$Fc$^+$C3$^+$ |
| New. | 67 | F | Spleen | MLL | 25 | 24 | 25 | 0 | <1 | 40 | | — | $\kappa$ | SIg$^+$Fc$^+$C3$^+$ |
| Tuf. | 67 | M | Node | ML Lp | 19 | 58 | 50 | NA | 0 | 68 | 0 | — | $\kappa$ | SIg$^+$Fc$^+$C3$^+$ |
| Smi. | 54 | M | Node | MLL | 10 | 32 | 39 | NA | 0 | 70 | 0 | — | $\mu\kappa$ | SIg$^+$Fc$^+$C3$^+$ |
| Holm. | 59 | F | Node | ML Lp | 8 | 25 | 65 | 11 | 0 | 80 | | — | $\gamma$ | SIg$^+$Fc$^+$C3$^+$ |
| Pat. | 67 | M | Marrow | MLL | 15 | 9 | 4 | 3 | 5 | 70 | | — | $\gamma\kappa$ | SIg$^+$ |
| Smi. | 62 | F | Node | MLL | 5 | 3 | 14 | 10 | 0 | 70 | | — | $\gamma$ | SIg$^+$ |
| Blo. | 57 | F | Node | ML Lp | 24 | 10 | 8 | 2 | 0 | 72 | 0 | — | $\kappa$ | SIg$^+$ |
| Jac. | 71 | M | Node | ML Lp | 6 | 2 | <1 | <1 | <1 | 38 | 0 | — | $\mu\kappa$ | SIg$^+$ |
| Bas. | 70 | M | Node | MLL | 6 | 7 | 3 | NA | 0 | 90 | | — | $\kappa$ | SIg$^+$ |
| Bes. | 62 | M | Node | MLL(A) | 12 | 12 | 13 | NA | NA | 30 | | — | ?$\mu\lambda$ | SIg$^+$ |
| Ber. | 55 | M | Node | ML Lp | 13 | 0 | 33 | NA | 5 | 70 | 0 | — | $\mu\kappa$ | SIg$^+$C3$^+$ |
| Smi. | 32 | F | Marrow | ML Lp | 43 | 6 | 36 | 0 | 0 | 47 | 9 | — | $\mu\lambda$ | SIg$^+$C3$^+$ |
| Tuc. (2) | 46 | F | Node | MLL | 17 | 17 | 69 | 17 | 0 | 50 | | — | $\mu\kappa$ | SIg$^+$C3$^+$ |
| Jac. | 53 | M | Node | ML Lp | 17 | 3 | 26 | 9 | 3 | 59 | 7 | — | $\mu\delta\kappa$ | SIg$^+$C3$^+$ |

*Note:* Where SIg staining was very weak, the figures of SIg$^+$ cells are given without regard to capping and non-capping.

TABLE VIII.—*Surface marker profile in various unclassified tumours*

| Patient | Age | Sex | Source | Histology | E | Fc | C3 | IgM | Phag | Caps | Non-caps | CyIg | Ig class | Profile |
|---|---|---|---|---|---|---|---|---|---|---|---|---|---|---|
| IqB. | 7 | M | Thymus | "Thymic blastoma" | 60 | 0 | 10 | 5 | 1 | 0 | 6 | — | — | T predominant |
| Hai. (1) | 35 | F | Node | Variant CB/cc | 48 | 16 | 32 | 0 | 10 | 25 | 8 | — | Pcl | T pred. Pcl B |
| Hai. (2) | 36 | F | Node | Variant CB/cc | 35 | 4 | 4 | 0 | 3 | 45 | 0 | — | μκ | SIg$^+$ |
| Cor. | 30 | F | Breast | Plasmacytoma/myeloma | 28 | 15 | 9 | 4 | 18 | 7 | 0 | 80 δλ | δλ | CyIg$^+$ |
| Tat. | 61 | F | Marrow | Waldenström's disease | 22 | 16 | 8 | 0 | 0 | 0 | 0 | {62 18} μκ | Pcl | CyIg$^+$ Polyclonal |
| Col. (2) | 48 | M | Marrow | ML Hg Pl | 74 | 32 | 38 | 40 | 0 | 17 | 0 | μλ | Pcl | E$^+$Fc$^+$IgM$^+$C3$^+$ |
| Umi. | 4 | M | Node | HMR | 10 | 3 | 13 | 3 | 2 | 24 | 0 | — | Pcl | Receptor-silent |
| Pea. (1) | 63 | M | Node | AILD | 70 | 0 | 0 | 0 | 1 | 3 | 0 | — | — | T predominant |
| Pea. (2) | 64 | M | Blood | MLIB | 90 | <1 | <1 | 0 | 0 | 3 | 1 | — | — | T predominant |

*Note:* *AILD = angio-immunoblastic lymphadenopathy with dysproteinaemia; HMR = histiocytic medullary reticulosis; MLIB = malignant lymphoma immunoblastic.

lar" in the Kiel classification. The same proportion of $SIg^+C3^+$ profiles (43%) were found in NPDL as in DPDL (40%) in this series, a finding at variance with those in some of the published work showing a higher proportion of nodular lymphomas with this profile.

*Surface markers in diffuse well differentiated malignant lymphoma (DWDL) (Table VII)*

DWDL was diagnosed in 17 patients contributing 19 profiles. In the cases of Tuc. (1, 2, 3), Bas., Smi., New., these lesions were associated with lymphocyte counts $>5\times10^9$/l. Most cases exhibited weak SIg staining after acetate washing, a feature constantly associated with tumours of this class, and with CLL. It was not possible in many cases to identify capping and non-capping cells because of weak SIg expression, which influences capping reactions. In several cases pre-acetate-wash cells exhibited strongly staining polyclonal Ig on the cell surface. Two patients (Tuc. (1) and Bas.) showed B cells expressing Fc only: this profile was encountered only in lymphomas of this histological class and in CLL, where Fc expression is always associated with IgM expression. In 6 patients the B cells exhibited $SIg^+Fc^+C3^+$ profile, which is not commonly found in other lymphomas. The spleen biopsy specimen (Tuc. (3)) exhibited $SIg^+Fc^+C3^+IgM^+$ profile, previously described in CLL. Six patients had $SIg^+$ cells only; in 2 patients (Blo., Jac.) SIg was expressed strongly, but only on 38% of cells in patient Jac. Three patients exhibited the profile $SIg^+C3^+$, a lower representation of this phenotype than in DPDL or NPDL tumours. All patients showed monoclonality of SIg after acetate wash, and the intensity of surface staining correlated with the Kiel histological classification. Of 8 biopsy specimens classified as "malignant lymphoma, lymphoplasmacytoid" (ML Lpc) strong SIg staining was seen in 7. Of the 11 biopsy specimens classified as "malignant lymphoma, lymphocytic" (MLL) only 1 (Smi.) showed strongly staining SIg. In several cases (those of Tuc. (2), Bes., Mar., New.) only a minority of cells expressed SIg, the major population being null. In no cases were more than 10% of cells found to have CyIg.

*Surface markers in non-classified tumours (Table VIII)*

Seven patients had lesions which did not qualify them for inclusion in any of the histological categories previously described. Patient IqB had a mediastinal tumour classified as "thymic blastoma", composed of epithelial and lymphoid elements of thymic derivation. The cells present were T-derived and the lesion classified at T-predominant, although the epithelial component did not react in any of the tests. Patient Hai. (1, 2) contributed 2 biopsy specimens of a unique lymphoma, thought to be a variant of centroblastic/centrocytic tumour of diffuse type. The patient Cor. had a plasmacytoma of breast expressing CyIg of DL class. The marrow and blood also contained neoplastic cells of this type. Patient Tat. had marrow involvement with Waldenström's disease and, unusually, showed a polyclonal population of cytoplasmic $IgM^+$ cells. In Patient Col. (2) the marrow examined 6 days after lymphnode biopsy (Col. (1) DPDL) showed a T-cell-predominant $Fc^+$, $C3^+$ and $IgM^+$ population. The cytology (ML Hg Pleomorphic) was quite unlike that of the lymphnode tumour (CB/cc/D). Patient Umi., with histiocytic medullary reticulosis, showed a receptor-silent profile on node cells. Patient Pea. presented with angio-immunoblastic lymphadenopathy with dysproteinaemia. The node profile suggested a T-cell neoplasm, but cytologically the predominant cell type was immunoblastic. The disease progressed to involve marrow and blood. The immunoblastic cytology persisted with $E^+$ cell markers. This is the second case of T-cell immunoblastic tumour in this series (see patient Ham., DH).

TABLE IX.—*Repeat biopsies in patients with NHL variations in histology and profile*

| Patient (Biopsy No.) | 1st Biopsy Rappaport | 1st Biopsy Kiel | 1st Biopsy Profile | 1st Biopsy SIg class | Interval (days) | 2nd Biopsy Rappaport | 2nd Biopsy Kiel | 2nd Biopsy Profile | 2nd Biopsy SIg class |
|---|---|---|---|---|---|---|---|---|---|
| Bre. (1) (2) | DU | MLLB(T) | E$^+$C3$^+$(IgM$^+$) | — | 275 | DU | MLLB(T) | E$^+$C3$^+$ | — |
| Mad (1) (2) | DU | HL Hg Pl | T predominant | Pcl | 7 | DU | MLLB | E$^-$HTLA$^+$ | — |
| Lov (1, 2) | DH | ML/cc Lc | T pred. Pcl B | Pcl | 361 | DH | ML/cc Lc | T predominant | — |
| Lov (2, 3) | DH | ML/cc Lc | T predominant | — | 361 | DH | ML/cc Lc | Receptor silent | — |
| Smi (1, 2) | DH | ML/CB | T pred. Mcl B | $\mu\kappa$ | 517 | DH | ML/CB$^+$IB | Non-capping B | $\gamma\mu\kappa$ |
| Has (1, 2) | NPDL | MLCB/cc/F | SIg$^+$C3$^+$ | $\mu\kappa$ | 399 | DH | MLIB | Non-capping B | $\mu\kappa$ |
| Kos (1, 2) | DPDL(A) | ML Lpc | SIg$^+$C3$^+$ | $\mu\kappa$ | 241 | DPDL(A) | ML Lg U | T pred. Mcl B | $\kappa$ |
| Kos (2, 3) | DPDL(A) | ML Lg U | T pred. Mcl B | $\kappa$ | 268 | DH | MLIB | Non-capping B | $\mu\kappa$ |
| Ted. (1, 2) | NPDL | CB/cc/F | Non-capping | $\mu\lambda$ | 572 | DH | MLCB/cc/CB/D | SIg$^+$ | $\mu\lambda$ |
| Ted. (2, 3) | NPDL | CB/cc/CB/D | SIg$^+$ | $\mu\lambda$ | 14 | NPDL | CB/cc/CB/D | SIg$^+$ | $\mu\lambda$ |
| Ted. (2, 3a) | NPDL | CB/cc/CB/D | SIg$^+$ | $\mu\lambda$ | 14 | NPDL | CB/cc/F | SIg$^+$CyIg$^+$ | $\mu\lambda^+\gamma\lambda$ |
| Bret. (1, 2) | NPDL | CB/cc/F | SIg$^+$C3$^+$ | Pcl | 169 | NPDL | CB/cc/F | SIg$^+$C3$^+$ | Pcl |
| Bri. (1, 2) | N+DPDL | CB/cc/F-D | SIg$^+$C3$^+$ | Pcl | 509 | M(L+H)D | CB/cc/D | SIg$^+$ | Pcl |
| Ayl. (1, 2) | NM(L+H) | CB/cc/F | SIg$^+$FC$^+$C3$^+$ | Pcl | 782 | NPDL | CB/cc/F$^+$Sc | SIg$^+$C3$^+$ | Pcl |
| Cha. (1, 2) | NPDL | CB/cc/F | SIg$^+$C3$^+$ | $\mu\lambda$ | 204 | N$^+$DPDL | CB/cc/F$^+$D | SIg$^+$C3$^+$ | $\mu\lambda$ |
| Pet (1, 2) | DPDL | ML/cc/Sc | SIg$^+$ | $\mu\lambda$ | 337 | DPDL | ML/cc/Sc | SIg$^+$ | $\mu\lambda$ |
| Eva. (1, 2) | NPDL | CB/cc/F | SIg$^+$C3$^+$ | $\gamma\kappa$ | 511 | NPDL | CB/cc/F | T pred. Mcl B | $\mu\kappa$ |
| Cou (1, 2) | NM(L+H) | CB/cc/F | T predominant | — | 119 | N+DPDL | CB/cc/F$^+$D | T pred. Mcl B | $\mu\kappa$ |
| Sul (1, 2) | NPDL | CB/cc/F | SIg$^+$C3$^+$ | Pcl | 348 | NPDL | CB/cc/F | SIg$^+$C3$^+$ | Pcl |
| Gig. (1, 2) | NPDL | CB/cc/F | T pred. Pcl B | Pcl | 604 | NPDL | CB/cc/F | SIg$^+$C3$^+$ | $\gamma\mu\lambda$ |
| Gig. (2, 3) | NPDL | CB/cc/F | SIg$^+$C3$^+$ | $\gamma\mu\lambda$ | 272 | NPDL | CB/cc/F | Non-capping | $\gamma\lambda$ |
| Tuc. (1, 2) | DWDL | MLL | SIg$^+$Fc$^+$ | $\mu$ | 340 | DWDL | MLL | SIg$^+$C3$^+$ | $\mu\kappa$ |
| Wil. (1, 2) | NPDL | CB/cc/F$^+$Sc | SIg$^+$C3$^+$ | $\gamma\kappa$ | 386 | NPDL | CB/cc/F$^+$Sc | SIg$^+$ | $\gamma\kappa$ |
| Wil. (2, 3) | NPDL | CB/cc/F$^+$Sc | SIg$^+$ | $\gamma\kappa$ | 162 | NPDL | CB/cc/F$^+$Sc | SIg$^+$ | Pcl |
| Hai. (1, 2) | Variant | CB/cc | T pred. Pcl B | Pcl | 212 | Variant | CB/cc/F | SIg$^+$ | $\mu\kappa$ |
| Pea. (1, 2) | AILD | — | T predominant | — | 720 | (Cytology, blood) | MLIB | T predominant | — |
| Col. (1) (2) | DPDL | CB/cc/D | SIg$^+$C3$^+$ | $\gamma\mu\kappa$ | 6 | (Marrow) | ML Hg Pl | E$^+$Fc$^+$C3$^+$IgM$^+$ | Pcl |

*Variations in profile and histology on repeat biopsy*

Changes in the histology of lymphomas during the course of the disease frequently involve conversion of a nodular lymphoma into diffuse lymphocytic or histiocytic lymphoma. In an attempt to document these changes, repeat biopsy specimens were examined in 23 patients. In patients Dep. (1, 2), Bro. (1, 2) and Tuc. (2, 3) lymphnode and spleen profiles were assessed concurrently, where laparotomy provided samples of involved spleen and node on the same day. These are not included in this section but are given in the appropriate Tables. In 2 of these patients the profile of tumour from node and spleen was the same (Dep. (1, 2), Bro. (1, 2)) while in Tuc. (2, 3) the node profile showed $SIg^+C3^+$ cells and the spleen profile $SIg^+Fc^+C3^+IgM^+$ cells.

In the remaining 21 patients, the histological variation and the change in surface profile, together with the interval between biopsies is given in Table IX. Four types of change occurred:

(1) *Changes in histological appearance.*—Nine patients (Mad., Smi., Has., Koz., Ted., Bri., Ayl., Cha., Cou.) showed changes. In Mad., the change was from a high-grade pleomorphic tumour into a malignant lymphoblastic lymphoma. This change was associated with loss of $E^+$ by the cells in this T-cell tumour, with reversion to an $E^-$ $HTLA^+$ phenotype. In patient Smi., the change from a malignant lymphoma of centroblastic type into a tumour containing both centroblasts and immunoblasts was accompanied by a change in profile from T predominance with monoclonal B cells into a non-capping lymphoma. In patient Has., the histological pattern changed from CB/cc/F (NPDL) to an immunoblastic malignant lymphoma (DH) accompanied by a change in phenotype from $SIg^+C3^+$ to a non-capping B-cell tumour. In patient Koz., 3 biopsy specimens showed marked histological progression from a lymphoplasmacytoid tumour to an unclassified low-grade malignant lymphoma to an immunoblastic lymphoma (DH). The profile altered during this sequence from $SIg^+C3^+$ to T-cell-predominant monoclonal B, and terminated in a non-capping B cell tumour. Patient Ted. underwent 4 biopsies (1, 2, 3, 3a). Nodes 3 and 3a were removed at one operation, 3 from the inguinal region, 3a from the axilla. All showed evidence of nodularity, but in contrast to biopsy specimen 1, biopsy specimens 2 and 3 showed some blast transformation with evidence of progression to a diffuse tumour (from CB/cc/F to CB/D). Biopsy specimen 3a showed preservation of the follicular appearance of biopsy specimen 1 (CB/cc/F). The phenotype of the node cells showed changes in the numbers of non-capping cells, and in T cells associated with these histological changes (Table VI). In patient Bri., the histology altered from a nodular and diffuse poorly differentiated tumour to a diffuse mixed lymphocytic and histiocytic lymphoma accompanied by a change in profile from $SIg^+C3^+$ to $SIg^+$. In both biopsy specimens SIg expression was polyclonal. In patient Ayl., the histological change was from nodular mixed lymphocytic and histiocytic lymphoma to a nodular poorly differentiated tumour with sclerosis. This was accompanied by a change in profile from $SIg^+Fc^+C3^+$ to $SIg^+C3^+$. In patient Cha., an alteration from a purely nodular tumour (NPDL; CB/cc/F) to a mixed nodular and diffuse tumour (N+DPDL, CB/cc/F+D) was not accompanied by any change in profile. In patient Cou., a nodular mixed lymphocytic and histiocytic tumour (CB/cc/F) progressed to a nodular and diffuse poorly differentiated lymphoma (CB/cc/F+D). This was accompanied by a change in profile from T-cell predominance to T-cell predominance with monoclonal B cells.

(2) *Changes in surface markers with no histological change.*—Patients Lov. (1, 2, 3), Eva. (1, 2), Gig. (1, 2, 3), Tuc. (1, 2), Wil. (1, 2), Hai. (1, 2) all showed changes in lymphnode profile, unaccompanied by changes in histology. In patient Lov., in 3 biopsies the histology showed diffuse histiocytic lymphoma (ML CC/Lc in Kiel classification). The profile changed from T predominance with polyclonal B cells to T predominance with no B cells, eventually becoming receptor-silent. The receptor-silent cells were not tested with HTLA antisera. In patient Eva., the profile changed from an $SIg^+C3^+$ tumour into a T-predominant tumour with monoclonal B cells without any alteration in histology. The SIg expressed by these cells changed from $\gamma\kappa$ to $\mu\kappa$. In patient Gig., in 3 biopsy specimens all showing nodular poorly differentiated histology (CB/cc/F), the profile changed from T-cell predominance with polyclonal B cells to $SIg^+C3^+$, and in the last biopsy specimen to a "slow capping" B-cell tumour. Alterations in SIg expression also occurred in this tumour. In patient Tuc., with diffuse well differentiated lymphoma of CLL type, the initial lymphnode biopsy specimen showed $SIg^+Fc^+$ B cells, while the second lymphnode biopsy showed $SIg^+C3^+$ B cells. In the first 2 biopsy specimens of patient Wil. (1, 2), both classified as NPDL, a change in profile from an $SIg^+C3^+$ tumour to an $SIg^+$ tumour occurred. In Hai., with a variant of centroblastic centrocytic lymphoma, the initial profile was of T-cell predominance with polyclonal B cells. The second biopsy specimen, of similar histological appearance, gave an $SIg^+$ B-cell profile.

(3) *Changes in expression of SIg.*—Patients Smi., Koz. (1, 2, 3), Ted., Eva., Cou., Gig. (1, 2, 3), Tuc. (1, 2), Wil. (2, 3) and Hai. all showed changes in SIg expression in the repeat biopsies. Patient Smi. showed a change from $\mu\lambda$ to $\gamma\mu\kappa$ with the appearance of $\gamma$ chain as well as $\mu$ on most cells in the second biopsy specimen. Patient Koz. showed an odd variation from $\mu\kappa$ monoclonality to expression of $\kappa$ only, followed by a reappearance of $\mu\kappa$ in the third biopsy specimen. In Patient Ted., the initial biopsy specimen was $\mu\lambda$ monoclonal, the last (3a) showed $\mu\lambda$ SIg with some $\gamma\lambda$ $CyIg^+$ cells. In Eva., the heavy-chain class altered from $\gamma$ to $\mu$ in a $\kappa$ chain-expressing tumour between the first and second biopsies. In Cou., no $SIg^+$ cells occurred in the first biopsy specimen, but $>10\%$ of cells in the second biopsy specimen were B cells and were $\mu\kappa$ monoclonal. In patient Gig., a B-cell component became predominant between the first and second biopsies and there was a change from polyclonal SIg expression to $\gamma\mu\lambda$ expression. Between the second and third biopsies, $\mu$ expression was lost, giving a $\gamma\lambda$ monoclonal B-cell tumour. In Patient Tuc., the first biopsy specimen showed only $\mu$ heavy chain on the B cells; the second biopsy showed $\mu\kappa$ monoclonality. In Patient Wil. (2, 3) a $\gamma\kappa$ monoclonal SIg profile in the second biopsy specimen had become polyclonal by the time of the third biopsy. In Patient Hai., a polyclonal B-cell component seen at the first biopsy expanded to a monoclonal B-cell component by the second biopsy, expressing $\mu\kappa$ SIg.

(4) *No change in histology or profile.*—Patients Bre., Bret., Pet., Sul., did not show changes either in histology or profile between the first and second biopsies. If allowance is made for the fact that the second biopsy in Bre. was done on cells from a malignant effusion, the profiles obtained (Table III) are probably equivalent, although in the node biopsy IgM receptor was present on a substantial proportion of T cells, and C3 was less well represented than in the pleural effusion. It is of interest that the SIg expression in both Bret. and Sul. remained polyclonal in both biopsy specimens, and no alteration in histology or profile was seen.

In Patients Pea. and Cal., changes in histology and profile are not comparable with those described in the other patients. In Patient Pea. the original biopsy specimen was not diagnosed as lymphoma. Only later, with the appearance of many circulating immunoblasts, was the progressive nature of the tumour appreciated. Profiles on blood, in our experience, are rarely interpretable in the same way as node or spleen profiles, and are probably the least representative of the involved tissues in non-Hodgkin lymphoma. In Patient Col.,

Table X.—*Percentage Ig expression in B lymphomas of different histological class*

| Class | Ig class expressed $\mu$ | $\gamma$ | $\alpha$ | $\kappa$ | $\lambda$ | $\gamma+\mu$ | Poly-clonal | $\kappa/\lambda$ ratio | H or L chain only |
|---|---|---|---|---|---|---|---|---|---|
| DU | 100 | 40 | 0 | 80 | 20 | 20 | 0 | 4·00 | 0 |
| DHL | 57 | 50 | 0 | 64 | 21 | 21 | 7 | 3·00 | 6 |
| DPDL | 75 | 38 | 4 | 38 | 50 | 21 | 8 | 0·76 | 7 |
| NPDL | 54 | 17 | 0 | 37 | 31 | 3 | 29 | 1·20 | 7 |
| DWDL | 53 | 16 | 0 | 74 | 16 | $\mu\delta\kappa$ 5 | 0 | 4·60 | 47 |

it is possible that 2 separate proliferating populations were initially present: B cells in lymphnode and T cells in marrow.

*Expression of SIg class in non-Hodgkin lymphomas of different histological type (Table X)*

The majority of neoplasms express $\mu$ heavy chain as the major immunoglobulin heavy-chain class. $\gamma$ Heavy chain is expressed with similar frequency in DU, DH, and DPDL lymphomas, but is rarely expressed in NPDL or DWDL lymphomas. Single-chain expression is unusual in DU, DH, DPDL or NPDL, but is very common in DWDL. In DHL, DU and DWDL, $\kappa$ light chain is expressed more frequently than $\lambda$; in DPDL $\lambda$ chain is expressed more frequently than $\kappa$. In NPDL the $\kappa/\lambda$ ratio is of the same order as in normal lymphnode cell suspensions. The number of B-cell-predominant polyclonal SIg$^+$ tumours is greater in NPDL lymphoma than in the other classes.

### DISCUSSION

1. *Relationship of surface marking to histology*

Of the many available classifications of non-Hodgkin lymphoma we chose to base this paper on two, the Rappaport scheme because of its proven clinical applicability, and the Kiel because it is one of the two major classifications embodying the Lukes and Collins follicular-centre-cell concept (Lukes & Collins, 1975; Gerard-Marchant *et al.*, 1974). Both classifications have strong and weak points: the Rappaport is simple to understand, and easy to apply. The major defect in the scheme lies in the mistaken concept of lymphocyte "differentiation" and in the division between histiocytic lymphomas (which are mainly lymphocytic) and lymphocytic lymphomas (Habeshaw *et al.*, 1977). The Kiel classification avoids these drawbacks, but is of a higher order of complexity and is largely unproven "in the field" of clinical applicability. In the first part of this discussion an attempt will be made to assess the validity of certain Kiel concepts, namely the ability to distinguish between B- and T-cell lineages cytologically, to identify follicular-centre-cell lesions, and to explore the Kiel division of the Rappaport "DWDL" lymphoma, into lymphocytic and lymphoplasmacytoid subgroups.

In the Kiel classification, lymphoblasts of B and T type are distinguished from those of ALL$^+$ type by cytology (convoluted nucleus, acid-phosphatase-positive T, cytoplasmic vacuolation and basophilia B). As shown in Table III, in 7 cases of T-lymphoblastic lymphoma proven by phenotype, 5 showed the cytological features of T lymphoblasts. Patients Leg. and Mad. did not show these features, and in both cases the lymphoblasts were E$^-$ and HTLA$^+$. These two cases may represent tumours of prethymic T lymphocytes (Kersey *et al.*, 1974). Of the 5 B-lymphoblastic lymphomas, 3 showed Burkitt-lymphoma-like features. Of interest is the presence of cytoplasmic immunoglobulin in 2 of these tumours, suggesting a relationship with the pre-B cell (SIg$^-$CyIgM$^+$ phenotype).

The Kiel classification stresses the interrelationship between centrocytes of large- and small-cell type, centroblasts, and immunoblasts as successive stages in the maturation of follicular lymphocytes to plasma cells. Lukes proposes a similar but not identical maturation sequence of small cleaved cell, large cleaved cell, small non-cleaved cell and large non-cleaved cell to the B-cell immunoblast. In terms of surface phenotype, normal follicular tissues show the presence of centrocytes of small- and large-cell type, which exhibit the $SIg^+C3d^+$ phenotype. Associated with these B cells are normally found a significant proportion of small and transformed cells (not centrocytes) which express E-rosetting capabilities, and are $HTLA^+$. In the tumours examined, most tumours containing $SIg^+C3d^+$ B cells had centroblastic and centrocytic morphology. However, of the 52 cases of centroblastic and centrocytic type, only 24 showed $SIg^+C3d^+$ phenotype. In 26 of these 52 cases, centroblastic and centrocytic tumours contained more than 20% of T lymphocytes, and some were T-lymphocyte-predominant. While there remains a good correlation between monoclonal B-cell-predominant tumours and the histological class "centroblastic and centrocytic" lymphoma, it is apparent that the B-cell subtype $SIg^+C3d^+$ is not detected in these tumours by morphology and cytology. The centroblastic component of the centroblastic and centrocytic tumour may well be T- rather than B-derived on the evidence of this series, because (1) 60% of purely centroblastic tumours were T-cell predominant; (2) centrocytic tumours of small- and large-cell type were B lymphoid; and (3) of the high incidence with a T-cell population $>20\%$ in tumours with a centroblastic component. If the "centroblast" is T-lymphoid, the relationship between the 2 centrocytic components and the immunoblast becomes easier to understand. The small-cell centrocytic tumours are frequently $SIg^+C3d^+$; some fail to cap SIg. The large-cell centrocytic tumours appear to lack C3d receptors, and are phenotypically similar to the B immunoblast (non-capping $SIg^+$). Small-cell centrocytic tumours and some centroblastic and centrocytic tumours have the phenotype $SIg^+$, which is also common in lymphoplasmacytoid malignancies. The latter can express $SIg^+C3d^+$, and $SIg^+Fc^+C3d^+$ phenotypes in addition. Our interpretation of the interrelationships of follicular lymphocyte subtypes in the Kiel classification would favour: (1) centroblast, and the non-transformed "small non-cleaved cell" of Lukes are T cells and represent the T-cell component associated with follicular B cells; (2) the small centrocyte ($SIg^+C3d^+$) transforming to the large centrocyte ($SIg^+$, non-capping) and to the immunoblast (non-capping $SIg^+$) or remaining untransformed, giving rise to the "lymphoplasmacytoid" lymphocyte ($SIg^+$, $SIg^+Fc^+C3d^+$). Immunoblasts of T type would, in this scheme, be derived from the centroblastic component of a centroblastic and centrocytic malignancy. Another prominent distinction between the Kiel and Rappaport classifications is the category of lymphoplasmacytoid malignancy in the Kiel classification, which forms a part of what Rappaport would call DWDL. On phenotypic grounds tumours of lymphoplasmacytoid type are quite clearly different from tumours of lymphocytic type. Lymphoplasmacytoid tumours exhibit capping SIg strongly, while the lymphocytic tumours show weak SIg expression, rather like the peripheral-blood lymphocyte in CLL. Although lymphoplasmacytoid tumours rarely contain more than 10% of $CyIg^+$ cells, some are always present, whilst in lymphocytic tumours plasma cells are very rare. From Table VII the correlation between strong SIg expression and lymphoplasmacytoid histology, and between weak SIg expression and lymphocytic histology is clearly shown.

In respect of the recognition of the classes of follicular-centre-cell derived tumours, the categorization morphologically and cytologically of T- and B-lymphoblastic tumours, and the distinction between the lymphoplasmacytoid and lymphocytic forms of diffuse lymphoma, the Kiel classification offers distinct advantages over the Rappaport scheme. The current limitations of histological and cytological classification using either Kiel or Rappaport are also apparent when the phenotypic correlations are taken into account. Neither scheme recognizes subtypes of B cell clearly defined by phenotype. Neither scheme achieves a one-for-one correlation of phenotype and morphology, even in a cytologically distinctive, monomorphic population. The conclusion clearly is that subsequent classifications of non-Hodgkin lymphoma must include surface marking as one of the diagnostic criteria.

When the correlations between surface marking and histology in the series of repeat biopsy specimens is examined, relationships between surface markers and histology become difficult to interpret. There is clear evidence of a relationship between T-cell-predominant monoclonal B-cell tumours, tumours of $SIg^+C3^+$ class and non-capping B-cell tumours of immunoblastic type. These sequences support the contention that tumours of follicular derivation are related to immunoblastic tumours. In terms of surface markers, there is strong evidence for believing that T-predominant monoclonal B-cell tumours and $SIg^+C3^+$ tumours are also functionally related, the end stage being either an $SIg^+$ non-capping or an $SIg^+$ tumour. The occurrence of T-cell predominance as one phase in the development of an $SIg^+C3^+$ or non-capping $SIg^+$ tumour has not previously been recognized. It is of importance, since in the normal immune response T cells accumulate in lymph nodes early, and their accumulation and division precede B-cell hyperplasia and antibody secretion (Davies *et al.*, 1969). Centroblastic tumours (although rare in this series) seem to correlate with the T-cell-predominant phase of what later becomes a B-cell-predominant tumour. Although the evidence is limited, we would like to suggest that the tumour of $SIg^+C3d^+$ phenotype is sandwiched between 2 T-cell-predominant phases, one of which precedes the expansion of the $SIg^+C3d^+$ clone, and the other which marks the transition between the $SIg^+C3d^+$ phenotype and the capping or non-capping $SIg^+$ tumour. During the first phase, the emergence of a follicular-cell pattern occurs, and during the second a change from a nodular to diffuse histological pattern is seen. Immunoglobulin switching from $\mu$ to $\gamma$ class, and the synthesis of CyIg probably occur after the second phase of T-cell predominance.

2. *Deductions about the nature of NHL from these studies*

Lymphomas are tumours of the immune system. The fact that they arise at all reflects some profound disturbance not just in the cell class termed "neoplastic" but in the precursors of that cell and in related cells which physiologically regulate the normal response to an antigen. A stem-cell defect in this system may be latent in both B- and T-cell lines, without compromising the function of either, until they meet the appropriate antigenic stimulus. If the defect is expressed in immunologically competent cells, and concerns events which occur after exposure of the cell to antigen, it will only become apparent as a failure of that cell to complete the immune response which would normally follow such exposure. In this sense, non-Hodgkin lymphoma (NHL) can be regarded as an abnormal immune response equivalent to a selective immune deficiency, limited to one clone of cells and evoked by a single antigen. Evidence for this statement is based on the close similarity between the chromosomal defect in ataxia telangiectasia and defects described in various forms of lymphoma (Louie & Schwartz, 1978; McCaw *et al.*, 1975; Fukahara *et al.*, 1976; Manolov & Manolova, 1972) and on the antibody activity of monoclonal immunoglobulins isolated from patients with lymphoma (Salmon & Seligmann, 1974).

Lymphomas can be broadly divided into two groups: (1) occurring before full functional differentiation of the lymphoid cells has been achieved and (2) tumours of differentiated lymphocytes. In Group 1 the tumours are of immunologically incompetent cells, and are independent of any antigenic stimulation. In Group 2 the tumours are of immunologically competent cells which are maturation-arrested at some stage in their response to an antigen. The defect (or oncogenic event) may be present in either tumour from the stem-cell stage, but in Group 1 tumours is expressed before the cell has achieved immunocompetence and in Group 2 tumours after functional differentiation is complete. Using this model it is possible to utilize the available evidence of normal differentiation to explore the nature of NHL.

Tumours of incompetent cells are the $ALL^+Ia^+$ lymphoblastic and the T-cell lymphoblastic lymphomas, including the $E^+C3^+$ subset. These tumours are usually $TdT^+$ (Kung *et al.*, 1978). It is during this phase of development that B and T cells acquire their antigen receptors by a process of proliferation, somatic mutation and elimination of self-antigen-reactive clones (Jerne, 1971). It has been suggested that TdT enzyme is important during the somatic mutation step in which the variable-region genes are assembled which code for the antigen-receptor sites on both the T-cell surface and the immunoglobulin molecule (Baltimore, 1974). The tumours of immunologically incompetent B cells are represented only by the pre-B cell ALL, since a measure of immunocompetence is present from the exhibition of SIg. All $SIg^+$ B-cell tumours are $TdT^-$ (Kung *et al.*, 1978; Donlon *et al.*, 1977; Habeshaw *et al.*, submitted for publication). Immature, but $SIg^+$ B cells have some immunocompetence. Their maturation can be blocked at this stage by anti-IgM antibody (Kearney *et al.*, 1976). After contact with anti-IgM, immature B cells shed their SIg and fail to secrete it again until the blocking agent is removed (Stocker, 1977; Sidman & Unanue, 1975). The effects of "blocking" B cells are reminiscent of the effects of removing polyclonal antibody from CLL cells by acetate washing, where only a weak or residual SIg positivity is retained, or in some cases no SIg expression at all. For this reason, the weakly staining CLL cells, and the malignant lymphomas of lymphocytic type may be tumours of immature B cells. It has been shown that Fc expression is an early characteristic of the B cell, preceding acquisition of the C3 receptor (Abbott *et al.*, 1976), and that $SIg^+Fc^+$ B cells require the presence of primed T cells and macrophages in order to respond to an antigen (Hoffman *et al.*, 1976). Virgin B cells, with $SIg^+Fc^+C3^+$ (and possibly $IgM^+$) phenotype, can respond to antigen in the presence of unprimed T cells and macrophages (Kearney *et al.*, 1978). The presence of weak SIg expression and $SIg^+Fc^+(IgM^+)$ phenotype as in CLL, might suggest tumours of immunocompetent but immature B cells. The profile of the virgin B cell is perhaps $SIg^+Fc^+C3^+IgM^+$, with surface IgM or $IgM^+D$ as the major SIg class (Coffman & Cohn, 1977). Most NHL are composed of B cells derived from the later phases of B-cell maturation. During germinal-centre development expansion and selection of antigen-reactive clones derived from marrow precursors occurs (Niewenhuis *et al.*, 1974; Niewenhuis & Keuning, 1974). The products of such selection are plasma-cell precursors and memory cells which, though derived from the same clone, probably belong to different maturation compartments (Zauderer & Askonas, 1976). Tumours of germinal-centre cells are represented by those of $SIg^+C3^+$ profile. Memory-cell tumours are of unknown profile, but since normal memory cells circulate and accumulate in the spleen (Niewenhuis & Keuning, 1974) their tumours may have $SIg^+Fc^+C3^+$ phenotype. According to one view (Hammerling *et al.*, 1976) the $SIg^+C3^+$ precursor from marrow has $SIg^+C3^-$ phenotype. T-cell help may be required for the differentiation of the $SIg^+C3^+$ B cell into a plasma-cell precursor (Lewis *et al.*, 1976). Pro-plasma cells do not recirculate and they probably express $SIg^+$ or $SIg^+CyIg^+$ profiles.

It is possible to identify those critical points in normal lymphocyte development at which tumours arise. These are (1) at stem-cell stage ($ALL^+Ia^+TdT^+$) or early in the differentiation sequence (pre-B, pre-thymic and thymic T cell); (2) between acquisition of SIg and virgin B-cell stage (as in MLL and CLL); (3) during germinal-centre formation ($SIg^+$ $C3^+$ tumours) in which there is at least one T-cell-predominant phase; or (4) during the change from $SIg^+$ to $CyIg^+$ cell, which may also include a T-cell-predominant phase and be accompanied by $IgM^-G$ switching and blast-cell transformation. The question how these maturation arrests occur should provide a useful challenge to the continuing investigation of non-Hodgkin lymphoma.

The authors would like to express their thanks to the following people: Members of the Childhood Cancer Study Group (Drs Mott, Willoughby, Graham-Pole, Gentle, Pritchard, Williams and others) who contributed biopsy material. Most of the cases were from the St Bartholomew's Hospital intake, and we are indebted to Drs T. A. Lister, J. S. Malpas, S. A. N. Johnson, R. Bell, J. Graham-Pole and Mr W. S. Shand for providing access to material. A number of people provided expert technical assistance, especially Geraldine Goodman, Merlin McGuire, and Susan Pegrum. Helpful advice and specific antisera were given by Drs M. F. Greaves, G. Janossy and Marion Roberts.

J.A.H. was in receipt of a Medical Research Council Training Fellowship for two years of this study.

### REFERENCES

ABBOTT, J., HOFFMANN, M. K., CHEN, A. F. & HAMMERLING, U. (1976) Sequential appearance of surface markers in the ontogeny of B lymphocytes. In *Progress in Differentiation Research*. Eds N. Müller-Bérat, C. Rosenfeld, D. Tarin & D. Viza. North Holland: Elsevier. p. 545.

BALTIMORE, D. (1974) Is terminal deoxynucleotidyl transferase a somatic mutagen in lymphocytes? *Nature*, **248**, 409.

BARRETT, S. G., SCHWADE, J. G., RANKEN, R. & KADIN, M. D. (1977) Lymphoblasts with both B and T markers in childhood leukaemia and lymphoma. *Blood*, **50**, 71.

BELPOMME, D., LE LARGE, N., MATHÉ, G. & DAVIES, A. J. (1977) Aetiological, clinical and prognostic significance of the T-B immunological classification of primary acute leukaemias and non-Hodgkin lymphomas. *Haematol. Bluttransfus.*, **20**, 17.

BOLHUIS, R. L. H. & NOOYEN, A. J. M. (1977) Receptors for IgM and IgM antigen complexes on human T lymphocytes reacting with specific anti-human T cell antiserum. *Immunology*, **33**, 679.

BRAYLAN, R. C., JAFFE, E. S., MANN, R. B., FRANK, M. M. & BERARD, C. W. (1977) Surface receptors of human neoplastic lymphoreticular cells. *Haematol. Bluttransfus.*, **20**, 47.

BRUNNING, R. D., MCKENNA, R. W., BLOOMFIELD, C. D., COCCIA, P. & GAJL-PECZALSKA, K. J. (1977) Bone marrow involvement in Burkitt's lymphoma. *Cancer*, **40**, 1771.

CHAPEL, H. M. & LING, N. R. (1977) Combined B and T lymphocyte marker test in lymphoproliferative disorders. *Br. J. Haematol.*, **35**, 367.

CHIAO, J. W., PANTIC, V. S. & GOOD, R. A. (1974) Human peripheral blood lymphocytes bearing both B cell complement receptors and T cell characteristics for sheep erythrocytes detected by a mixed rosette method. *Clin. Exp. Immunol.*, **18**, 483.

COFFMAN, R. L. & COHN, M. (1977) The class of surface immunoglobulin on virgin and memory B lymphocytes. *J. Immunol.*, **118**, 1806.

DAVIES, A. J. S., CARTER, R. L., LEUCHARS, E., WALLACE, V. & KOLLER, P. C. (1969) The morphology of immune reactions in normal, thymectomised and reconstituted mice. I—The response to sheep erythrocytes. *Immunology*, **16**, 57.

DICKLER, H. B., ADKINSON, N. F. & TERRY, W. D. (1974) Evidence for individual human peripheral blood lymphocytes bearing both B and T cell markers. *Nature* (*New Biol.*), **247**, 213.

DONLON, J. A., JAFFE, E. S. & BRAYLAN, R. C. (1977) Terminal deoxynucleotidyl transferase activity in malignant lymphomas. *N. Engl. J. Med.*, **297**, 461.

FERRARINI, M., MORETTA, L., ABRILE, R. & DURANTE, M. L. (1975) Receptors for IgG molecules on human lymphocytes forming spontaneous rosettes with sheep cells. *Eur. J. Immunol.*, **5**, 70.

FUKAHARA, S., SHIRAKAWA, S. & UCHINO, H. (1976) Specific marker chromosome 14 in malignant lymphomas. *Nature*, **259**, 210.

GERARD-MARCHANT, R., HAMBLIN, I., LENNERT, K., RILKE, F., STANSFELD, A. G. & VAN UNNIK, J. A. M. (1974) Classification of non-Hodgkin lymphomas. *Lancet*, ii, 406.

GREAVES, M. F., JANOSSY, G., ROBERTS, M. & 5 others (1977) Membrane phenotyping, diagnosis, monitoring and classification of acute "lymphoid" leukaemias. *Haematol. Bluttransfus.*, **20**, 61.

GREAVES, M. F., WILLIAMS, R. C. & SEYMOUR, G. J. (1978) Assays for human lymphocyte subpopulations. In *Current Research in Rheumatoid Arthritis and Allied Diseases*. Eds D. C. Dumonde & R. M. Maini. London: Medical & Technical Press.

HABESHAW, J. A., MACAULAY, R. A. A. & STUART, A. E. (1977) Correlation of surface receptors with histological appearance in 29 cases of non-Hodgkin lymphoma. *Br. J. Cancer*, **35**, 858.

HABESHAW, J. A. & STUART, A. E. (1975) Cell receptor studies on seven cases of diffuse histiocytic malignant lymphoma (reticulum cell sarcoma). *J. Clin. Pathol.*, **28**, 289.

HABESHAW, J. A., STUART, A. E., DEWAR, A. E. & YOUNG, G. A. (1976) IgM receptors on T cells in Hodgkin's disease. (Letter). *Lancet*, i, 916.

HAMMERLING, U., CHEN, A. F. & ABBOTT, J. (1976) The ontogeny of murine B lymphocytes. II—The sequence of B cell differentiation from surface Ig negative precursors to plasma cells. *Proc. Natl Acad. Sci. U.S.A.*, **73**, 2008.

HANN, H. L., LONDON, W. T. & EVANS, A. E. (1977) Lymphoblasts with T cell markers in 5 girls with acute lymphocytic leukaemia. *Cancer*, **39**, 2001.

HARRIS, N. S. (1974) Plasma cell surface antigen on human blood lymphocytes. *Nature*, **250**, 507.

HOFFBRAND, A. V., GANESHAGURU, K., JANOSSY, G., GREAVES, M. F., CATOVSKY, D. & WOODRUFF, R. K. (1977) Terminal deoxynucleotidyl transferase levels and membrane phenotypes in diagnosis of acute leukaemia. *Lancet*, ii, 520.

HOFFMAN, M. K., HAMMERLING, U., SIMON, M. & OETTGEN, H. F. (1976) Macrophage requirements of $CR^-$ and $CR^+$ B lymphocytes for antibody production *in vitro*. *J. Immunol.*, **116**, 1447.

HUTTEROTH, T. H., LITWIN, S. D. & CLEVE, H. (1972) Cultured lymphoid cell lines from normal subjects: membrane associated immunoglobulins studied by the mixed antiglobulin reaction. *Cell. Immunol.*, **5**, 446.

JAFFE, E. S., SHEVACH, E. M., FRANK, M. M., BERARD, C. & GREEN, I. (1974) Nodular lymphoma—evidence for origin from follicular B lymphocytes. *N. Engl. J. Med.*, **290**, 813.

JAFFE, E. S., BRAYLAN, R. C., NANBA, K., FRANK, M. M. & BERARD, C. W. (1977) Functional markers: a new perspective on malignant lymphomas. *Cancer Treat. Rep.*, **61**, 953.

JANOSSY, G., GOLDSTONE, A. H., CAPELLARO, D. & 4 others (1977) Differentiation-linked expression of p 28 33 (Ia-like) structures on human leukaemic cells. *Br. J. Haematol.*, **37**, 391.

JERNE, N. K. (1971) The somatic generation of immune recognition. *Eur. J. Immunol.*, **1**, 1.

KABAT, E. A. & MAYER, M. M. (1961) In *Experimental Immunochemistry*, 2nd edn. Springfield: Charles C. Thomas. p. 150.

KEARNEY, J. F., COOPER, M. D. & LAWTON, A. R. (1976) B lymphocyte differentiation induced by lipopolysaccharide. III—Suppression of B cell maturation by anti-mouse immunoglobulin antibodies. *J. Immunol.*, **116**, 1664.

KEARNEY, J. F., KLEIN, J., BOCKMAN, D. E., COOPER, M. D. & LAWTON, A. R. (1978) B cell differentiation induced by lipopolysaccharide. V—Suppression of plasma cell maturation by anti-$\mu$. Mode of action and characteristics of suppressed cells. *J. Immunol.*, **120**, 158.

KERSEY, J. H., NESBIT, M. E., LUCKASEN, J. R. & 4 others (1974) Acute lymphoblastic leukaemia and lymphoma cells with thymus derived (T) markers. *Mayo Clin. Proc.*, **49**, 584.

KUNG, P. C., LONG, J. C., MCCAFFREY, R. P., RATCLIFF, R. L., HARRISON, T. A. & BALTIMORE, D. (1978) Terminal deoxynucleotidyl transferase in the diagnosis of leukaemia and lymphoma. *Am. J. Med.*, **64**, 788.

LACHMANN, P. J., HOBART, M. J. & ASTON, W. P. (1973) Complement technology. In *Handbook of Experimental Immunology*. Ed. D. M. Weir. Oxford: Blackwell Scientific Pub. p. 8.

LEWIS, G. K., RANKEN, R., NITECKI, D. E. & GOODMAN, J. W. (1976) Murine B cell subpopulations reponsive to T dependent and T independent antigens. *J. Exp. Med.*, **144**, 382.

LOUIE, S. & SCHWARTZ, R. S. (1978) Immunodeficiency and the pathogenesis of lymphoma and leukaemia. *Semin. Haematol.*, **15**, 117.

LUKES, R. J. & COLLINS, R. D. (1974) Immunological characterisation of human malignant lymphomas. *Cancer*, **34**, 1488.

LUKES, R. J. & COLLINS, R. D. (1975) New approaches to the classification of the lymphomata. *Br. J. Cancer*, **31**, Suppl. 2, 1.

MCCAFFREY, R., HARRISON, T. A., PARKMAN, R. & BALTIMORE, D. (1975) Terminal deoxynucleotidyl transferase activity in human leukaemic cells and in normal thymocytes. *N. Engl. J. Med.*, **292**, 775.

MCCAW, B. K., HECHT, F. & HARNDEN, D. G. (1975) Somatic rearrangement of chromosome 14 in human lymphocytes. *Proc. Natl Acad. Sci. U.S.A.*, **72**, 2071.

MCCONNELL, I. & HURD, C. M. (1976) Lymphocyte receptors. II—Receptors for rabbit IgM on human T lymphocytes. *Immunology*, **30**, 835.

MANOLOV, G. & MANOLOVA, Y. (1972) Marker band in one chromosome 14 from Burkitt lymphomas. *Nature*, **237**, 33.

MORETTA, L., MINGARI, M. C., MORETTA, A. & LYDYARD, P. M. (1977) Receptors for IgM are expressed on acute lymphoblastic leukaemic cells having T cell characteristics. *Clin. Immunol. Immunopathol.*, **7**, 405.

NIEWENHUIS, P. & KEUNING, F. J. (1974) Germinal centres and the origin of the B cell system. II—Germinal centres in the rabbit spleen and popliteal lymph nodes. *Immunology*, **26**, 509.

NIEWENHUIS, P., VAN-NOUHUIJS, C. E., EGGENS, J. H. & KEUNING, F. J. (1974) Germinal centres and the origin of the B cell system. I—Germinal centres in the rabbit appendix. *Immunology*, **26**, 497.

PEARL, E. R., VOGLER, L. B., OKOS, A. J., CRIST, W. M., LAWTON, A. R. & COOPER, M. D. (1978) B lymphocyte precursors in human bone marrow: an analysis of normal individuals and patients with antibody deficiency states. *J. Immunol.*, **120**, 1169.

PILCHER, W. J. & KNAPP, W. (1977) Binding of IgM coated erythrocytes to chronic lymphocytic leukaemia (CLL) cells. *Scand. J. Immunol.*, **6**, 736.

ROBERTS, M., GREAVES, M., JANOSSY, G., SUTHERLAND, R. & PAIN, C. (1978) Acute lymphoblastic leukaemia (ALL) associated antigen—I. Expression in different haematopoietic malignancies. *Leukaemia Res.*, **2**, 105.

SALMON, S. E. & SELIGMANN, M. (1974) B cell neoplasia in man. *Lancet*, ii, 1230.

SELIGMANN, M. (1974) B cell and T cell markers in lymphoid proliferations. *N. Engl. J. Med.*, **290**, 753.

SIDMAN, C. L. & UNANUE, E. R. (1975) Receptor mediated inactivation of early B lymphocytes. *Nature*, **257**, 149.

STEIN, H. (1976) Immunochemische und immunozytologische Befund bei non-Hodgkin Lymphomen. *Haematol. Bluttransfus.*, **18**, 167.

STEIN, H., PETERSEN, N., GAEDICKE, G., LENNERT, K. & LANDBECK, G. (1976) Lymphoblastic lymphoma of convoluted or acid phosphatase type, a tumour of T precursor cells. *Int. J. Cancer*, **17**, 292.

STEIN, H., SIEMSSEN, U. & LENNERT, K. (1978) Complement receptor subtypes C3b and C3d in lymphatic tissue and follicular lymphomas. *Br. J. Cancer*, **37**, 520.

STOCKER, J. W. (1977) Tolerance induction in maturing B cells. *Immunology*, **32**, 283.

STUART, A. E. & HABESHAW, J. A. (1976) Receptor studies on 19 cases of non-Hodgkin malignant lymphocytic lymphoma. *Acta Haematol.*, **55**, 160.

TOBEN, H. R. & SMITH, R. G. (1977) T lymphocytes bearing complement receptors in a patient with chronic lymphocytic leukaemia. *Clin. Exp. Immunol.*, **27**, 292.

VOGLER, L. B., CRIST, W. M., BOCKMAN, D. E., PEARL, E. R., LAWTON, A. R. & COOPER, M. D. (1978) Pre-B cell leukaemia. A new phenotype of childhood lymphoblastic leukaemia. *N. Engl. J. Med.*, **298**, 872.

WERNET, P. & WILMS, K. (1977) Human Ia alloantigens as cell differentiation markers of normal and pathological leucocyte surfaces. *Scand. J. Immunol.*, **6**, 563.

ZAUDERER, M. & ASKONAS, B. A. (1976) Several proliferative phases precede maturation of IgG secreting cells in mitogen stimulated cultures. *Nature*, **260**, 611.